# Medical contrast agents as promising tools for biomacromolecular SAXS experiments

**DOI:** 10.1107/S2059798322007392

**Published:** 2022-08-09

**Authors:** Frank Gabel, Sylvain Engilberge, Emmanuelle Schmitt, Aurélien Thureau, Yves Mechulam, Javier Pérez, Eric Girard

**Affiliations:** a IBS, CEA, CNRS, UGA, 71 Avenue des Martyrs, 38000 Grenoble, France; b IOC, Ecole polytechnique, CNRS, Institut Polytechnique de Paris, 91128 Palaiseau, France; c Synchrotron SOLEIL, Saint-Aubin BP 48, 91192 Gif-sur-Yvette, France; Station Biologique de Roscoff, France

**Keywords:** small-angle scattering, SAXS, contrast variation, macromolecular complexes, electron density, medical contrast agents, iohexol, Gd-HPDO3A, tools for SAXS

## Abstract

Small-angle X-ray scattering (SAXS) has become an indispensable tool in structural biology, complementing atomic-resolution techniques. It is sensitive to the electron-density difference between solubilized biomacromolecules and the buffer, and provides information on molecular masses, particle dimensions and interactions, low-resolution conformations and pair distance-distribution functions. When SAXS data are recorded at multiple contrasts, *i.e.* at different solvent electron densities, it is possible to probe, in addition to their overall shape, the internal electron-density profile of biomacromolecular assemblies. Unfortunately, contrast-variation SAXS has been limited by the range of solvent electron densities attainable using conventional co-solutes (for example sugars, glycerol and salt) and by the fact that some biological systems are destabilized in their presence. Here, SAXS contrast data from an oligomeric protein and a protein–RNA complex are presented in the presence of iohexol and Gd-HPDO3A, two electron-rich molecules that are used in biomedical imaging and that belong to the families of iodinated and lanthanide-based complexes, respectively. Moderate concentrations of both molecules allowed solvent electron densities matching those of proteins to be attained. While iohexol yielded higher solvent electron densities (per mole), it interacted specifically with the oligomeric protein and precipitated the protein–RNA complex. Gd-HPDO3A, while less efficient (per mole), did not disrupt the structural integrity of either system, and atomic models could be compared with the SAXS data. Due to their elevated solubility and electron density, their chemical inertness, as well as the possibility of altering their physico-chemical properties, lanthanide-based complexes represent a class of molecules with promising potential for contrast-variation SAXS experiments on diverse biomacromolecular systems.

## Abstract 2

Lanthanide-based complexes are presented as a promising class of molecules for efficient SAXS contrast-variation experiments. Their interactions and contrast properties are analyzed for an oligomeric protein and a protein–RNA complex.

## 1. Introduction

Biological small-angle X-ray scattering (BioSAXS) has been used for several decades to extract structural information from a multitude of systems in aqueous solution, including protein–RNA/DNA complexes, solubilized membrane proteins and viruses (Glatter, 2018 ▸; Svergun *et al.*, 2013 ▸; Putnam *et al.*, 2007 ▸; Lipfert & Doniach, 2007 ▸; Lindner & Zemb, 2002 ▸). It is sensitive to the electron-density difference Δρ between the solubilized particles (ρ) and the solvent (ρ_sol_), and provides structural information in the nanometre to micrometre range. While BioSAXS has witnessed significant progress in instrumentation and sample environment, as well as in automation of data processing and analysis (Thureau *et al.*, 2021 ▸; Jeffries *et al.*, 2021 ▸; Brosey & Tainer, 2019 ▸; Hajizadeh *et al.*, 2018 ▸; Tuukkanen *et al.*, 2017 ▸), the use of solvent contrast variation (*i.e.* changing ρ_sol_) is largely underdeveloped. Indeed, most BioSAXS experiments are carried out with a single contrast/solvent (typically aqueous buffers containing small amounts of salt and molecules to adjust the pH). Under these conditions, it is difficult to accurately probe the internal electron-density distribution of complex biomacromolecular assemblies (*i.e.* particles composed of segregated zones of different electron density, for example membrane proteins and protein–RNA/DNA complexes).

A detailed analysis of the internal topology of such biomacromolecular complexes is routinely performed in small-angle neutron scattering (SANS) by varying the solvent scattering length density (SLD) by H_2_O/D_2_O exchange (Mahieu & Gabel, 2018 ▸), a procedure that is capable of covering the full range of SLD of all major biomolecules (proteins, RNA/DNA, lipids and detergents). Solvent-variation approaches have less often been employed in SAXS for several practical reasons: firstly, the capacities of ‘conventional’ molecules [for example, sucrose (Garcia-Diez *et al.*, 2016 ▸; Bolze *et al.*, 2003 ▸; Kiselev *et al.*, 2001 ▸; Ballauff, 2001 ▸; Dingenouts & Ballauff, 1993 ▸), glycerol (Hickl *et al.*, 1996 ▸; Bolze *et al.*, 1996 ▸) and salt (Naruse *et al.*, 2009 ▸; Fernandez *et al.*, 2008 ▸)] to alter the electron density of a buffer are limited. The highest values reported in early work included 0.43 e^−^ Å^−3^ for 2.5 *M* sucrose in a myoglobin study (Stuhrmann, 1970 ▸) and 0.446 e^−^ Å^−3^ for 7.0 *M* NaBr in a low-density lipid (LDL) protein study (Aggerbeck *et al.*, 1978 ▸). In the majority of cases ρ_sol_ is varied between 0.34 (pure water) and a maximum of ∼0.42 e^−^ Å^−3^, which is reached for 2.0 *M* [55%(*w*/*w*)] sucrose (Jeffries *et al.*, 2016 ▸; Kiselev *et al.*, 2001 ▸, 2003 ▸; Dingenouts & Ballauff, 1993 ▸; Kirste & Stuhrmann, 1967 ▸) or 100% glycerol (Wolf *et al.*, 1989 ▸; Kirste & Stuhrmann, 1967 ▸). The values thus obtained correspond to the lower range of protein electron densities (Jeffries *et al.*, 2016 ▸; ∼0.42–0.44 e^−^ Å^−3^), but are far below those of RNA/DNA (Feigin & Svergun, 1987 ▸; ∼0.55 e^−^ Å^−3^) or lipid and detergent head groups (Breyton *et al.*, 2013 ▸; ∼0.52 e^−^ Å^−3^). Secondly, conventional contrast agents, in particular salt solutions, have been reported to alter the structural integrity of biomacromolecular assemblies (Chen *et al.*, 2017 ▸), which is possibly the reason why very concentrated NaBr solutions, as reported in the early LDL study, have not been further used with other biological systems. NaCl solutions can provide electron densities of up to 0.38 e^−^ Å^−3^ (at 5 *M*; Wolf *et al.*, 1989 ▸), but are equally problematic since they may perturb the structural integrity of several systems (Chen *et al.*, 2017 ▸; Naruse *et al.*, 2009 ▸; Fernandez *et al.*, 2008 ▸). Finally, conventional contrast agents cannot be ‘improved’ in the sense that they have a fixed chemical structure and their capacity to alter solvent electron densities depends solely on their concentration.

In the present study, we demonstrate that a certain class of chemically more versatile molecules have promising potential to serve as efficient SAXS contrast agents. For this, we applied two electron-rich medical contrast media, iohexol and Gd-HPDO3A, in SAXS experiments on a hexameric protein and a protein–RNA complex: protease 1 from *Pyrococcus horikoshii* (Engilberge *et al.*, 2017 ▸) and the aIF2:GDPNP:methionylated initiator tRNA complex from *Saccharolobus solfataricus* (Schmitt *et al.*, 2012 ▸). Iohexol (C_19_H_26_I_3_N_3_O_9_), commercialized under the trade names Omnipaque, Histodenz and Nycodenz (among others), is a non-ionic tri-iodinated molecule that is used as a medical X-ray imaging contrast medium. Gd-HPDO3A (C_17_H_29_GdN_4_O_7_) or gadoteridol (trade name ProHance) is a neutral lanthanide complex that is used as a paramagnetic contrast agent for magnetic resonance imaging (MRI) and contains a single Gd atom surrounded by an HPDO3A macrocycle. Concentrated solutions of both media readily attained electron densities of 0.44 e^−^ Å^−3^, equivalent to the upper range of typical protein densities. While iohexol was more efficient (per mole) in increasing the solvent density, it displayed a strong preferential interaction with the protein and led to aggregation of the protein–RNA complex. Gd-HPDO3A, on the other hand, did not perturb the structure of the oligomeric protease 1 or of the protein–RNA complex and a detailed comparison with atomic models was possible.

## 2. Materials and methods: summary

Iohexol {IUPAC name *N*1,*N*3-bis(2,3-dihydroxypropyl)-5-[*N*-(2,3-dihydroxypropyl)acetamido]-2,4,6-triiodobenzene-1,3-dicarboxamide} was purchased from Sigma–Aldrich (catalog No. D2158). Gd-HPDO3A was kindly provided by Bracco Imaging S.p.A., Milan, Italy. Contrast-agent stock solutions were prepared in Milli-Q water. Appropriate amounts of the contrast-agent stock solutions were lyophilized and resolubilized with appropriate volumes of buffer, protein and protein–RNA stock solutions to obtain the concentration series measured by SAXS. Solvent electron densities were calculated from the amounts of contrast agents and buffer added and the final volumes of the stock solutions.

aIF2γα (domain 3) and 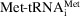 (A1–U72) were prepared as described by Monestier *et al.* (2017 ▸) and Schmitt *et al.* (2012 ▸). Protein and tRNA were mixed in a 1:1.2 molar ratio in the presence of 1 m*M* GDPNP and 5 m*M* MgCl_2_. The protein–tRNA complex was then purified by molecular sieving and concentrated to 5 mg ml^−1^ in 10 m*M* MOPS–NaOH pH 6.7, 200 m*M* NaCl, 5 m*M* MgCl_2_, 1 m*M* GDPNP. Protease 1 from *Pyrococcus horikoshii* was purified following existing protocols (Engilberge *et al.*, 2017 ▸) and concentrated to obtain an 8.4 mg ml^−1^ stock solution in aqueous buffer (20 m*M* Tris pH 7.5, 150 m*M* NaCl).

All SAXS experiments were carried out on the SWING beamline (https://www.synchrotron-soleil.fr/en/beamlines/swing) at the SOLEIL synchrotron, Saint-Aubin, France in flow mode using X-ray energies of 12.00 or 14.00 keV and sample-to-detector distances of 1.79 or 2.00 m. For each sample, a volume of 40 µl was circulated at 75 µl min^−1^ through a thermalized quartz capillary of 1.5 mm diameter and 10 µm wall thickness inserted within a vacuum chamber (David & Pérez, 2009 ▸). Series of individual 0.5 or 1 s time frames (typically between 5 and 30) were collected at 15°C. The 2D scattering patterns were reduced to 1D intensities and binned using the *Foxtrot* software (Thureau *et al.*, 2021 ▸) after manually checking for radiation damage and validation of identical transmissions. Buffer intensities were subtracted from sample intensities using *PRIMUS* (Manalastas-Cantos *et al.*, 2021 ▸) after careful calibration against the measured transmissions following a previously established protocol (Gabel *et al.*, 2019 ▸): briefly, the contrast-agent concentrations in both the sample and the buffer solutions were calibrated against a reference curve and the buffers were corrected to match the sample concentrations by using an approximation of a local linear interpolation between adjacent data points.

Basic biomacromolecular parameters [radii of gyration *R*_g_, pair distance distribution functions *p*(*r*) and maximum dimensions *D*_max_] were determined using *PRIMUS* and *GNOM* (Svergun, 1992 ▸). Partial specific volumes (used to calculate theoretical match points) were taken from the literature (Voss & Gerstein, 2005 ▸; Kharakoz, 1997 ▸; Creighton, 1993 ▸; Jacrot, 1976 ▸). The scattering patterns from the free Gd-HPDO3A molecules were fitted by the combination of a spherical form factor with a modified hard-sphere interaction potential (Guinier & Fournet, 1955 ▸; Fournet, 1951 ▸). Preferential binding of iohexol was analyzed by three complementary approaches: fitting of atomic models with *CRYSOL* (Svergun *et al.*, 1995 ▸), relative changes of forward-scattered intensities *I*(0) and the experimental values of contrast-match points (CMPs).

Full details of sample preparation and SAXS data reduction/analysis are included in the supporting information.

## 3. Results

### 3.1. Gd-HPDO3A is an efficient, inert SAXS contrast agent

**Figure 1:**
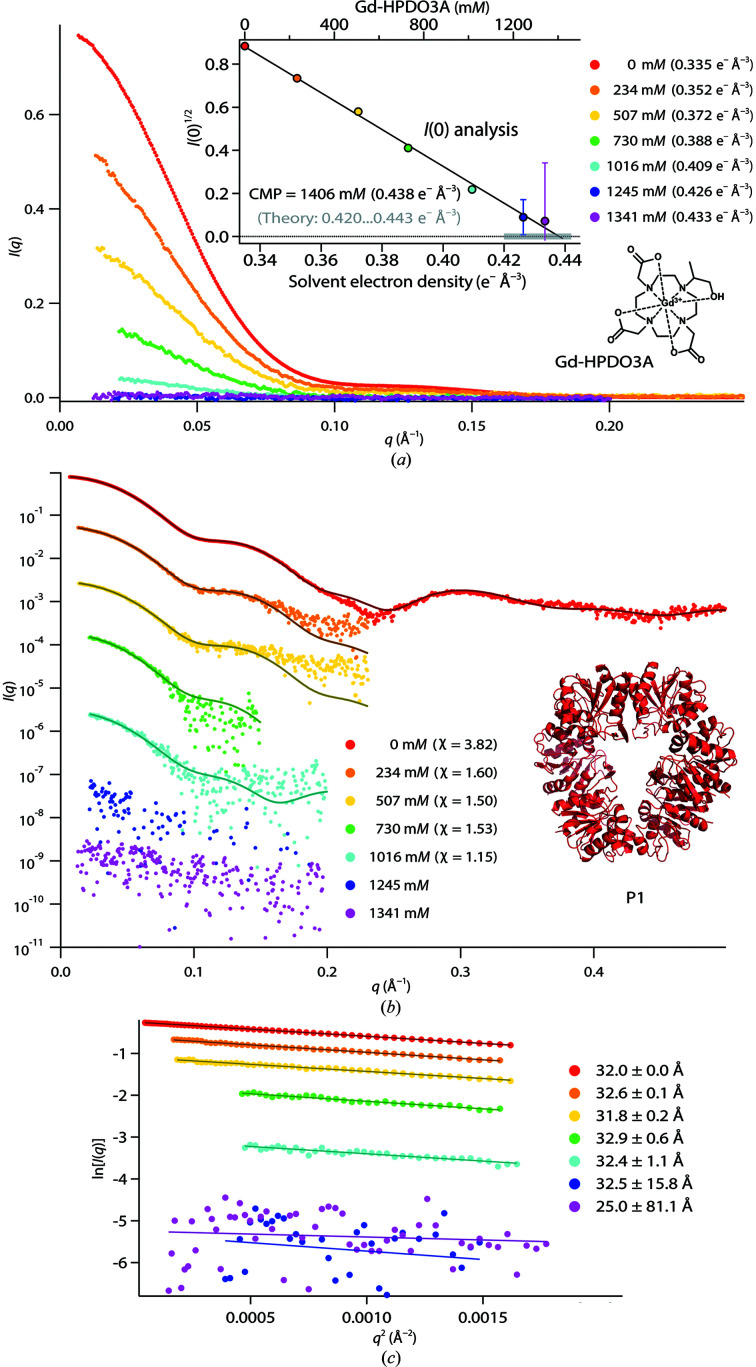
P1 SAXS data with Gd-HPDO3A. (*a*) SAXS curves (linear scale) at various Gd-HPDO3A concentrations. Insets: the square root of the scattering intensity at zero angle, *I*(0)^1/2^, and linear fit for determination of the CMP; chemical formula of Gd-HPDO3A. (*b*) *CRYSOL* fits with a hexameric P1 model (PDB entry 7qo8; see supporting information), logarithmic scale. For reasons of legibility, the different SAXS curves were shifted. (*c*) Guinier fits used to determine the *R*_g_ and *I*(0) values.

We made SAXS measurements of the hexameric protein protease 1 (hereafter abbreviated P1) from *P. horikoshii* and the aIF2γα (domain 3):GDPNP:Met-tRNA_i_^Met^ complex (aIF2-tRNA) from *S. solfataricus* in Gd-HPDO3A solutions of up to 1.38 *M* (Figs. 1 ▸ and 2 ▸).

The scattered signal of P1 was virtually masked at the maximum Gd-HPDO3A concentration (1341 m*M*; Fig. 1*a*, pink points), indicating that the contrast-match point (CMP) of the protein was practically reached at the highest solvent electron density (0.433 e^−^ Å^−3^). This result was confirmed by determination of the CMP from a linear fit of *I*(0)^1/2^ versus the Gd-HPDO3A concentration (Fig. 1 ▸ *a*, inset). Importantly, the experimental CMP (0.438 e^−^ Å^−3^) was well within the range predicted from the protein sequence (0.420–0.443 e^−^ Å^−3^; Supplementary Table S1) and the linearity of *I*(0)^1/2^ indicates that the oligomeric state of P1 was preserved at all Gd-HPDO3A concentrations. Experimental SAXS curves were fitted with a hexameric atomic model (PDB entry 7qo8, see supporting information) using *CRYSOL* and imposing a fixed, calculated, electron density for the bulk solvent (see the supporting information for details). These fits showed good agreements at all Gd-HPDO3A concentrations up to 1016 m*M* (Fig. 1 ▸ *b*). Moreover, the experimental radii of gyration (*R*_g_) did not vary significantly throughout the series (Fig. 1 ▸ *c*). (Note that the *R*_g_ values at the two highest Gd-HPDO3A concentrations depended strongly on the Guinier fit range and have large error bars due to the weak contrast.)

Altogether, the P1 data suggest that Gd-HPDO3A molecules are inert towards this protein and their presence (at up to molar concentrations) does not alter either the conformation of individual monomers or the overall oligomeric state and does not generate any appreciable solvation effects or specific interactions with the protein surface.

**Figure 2:**
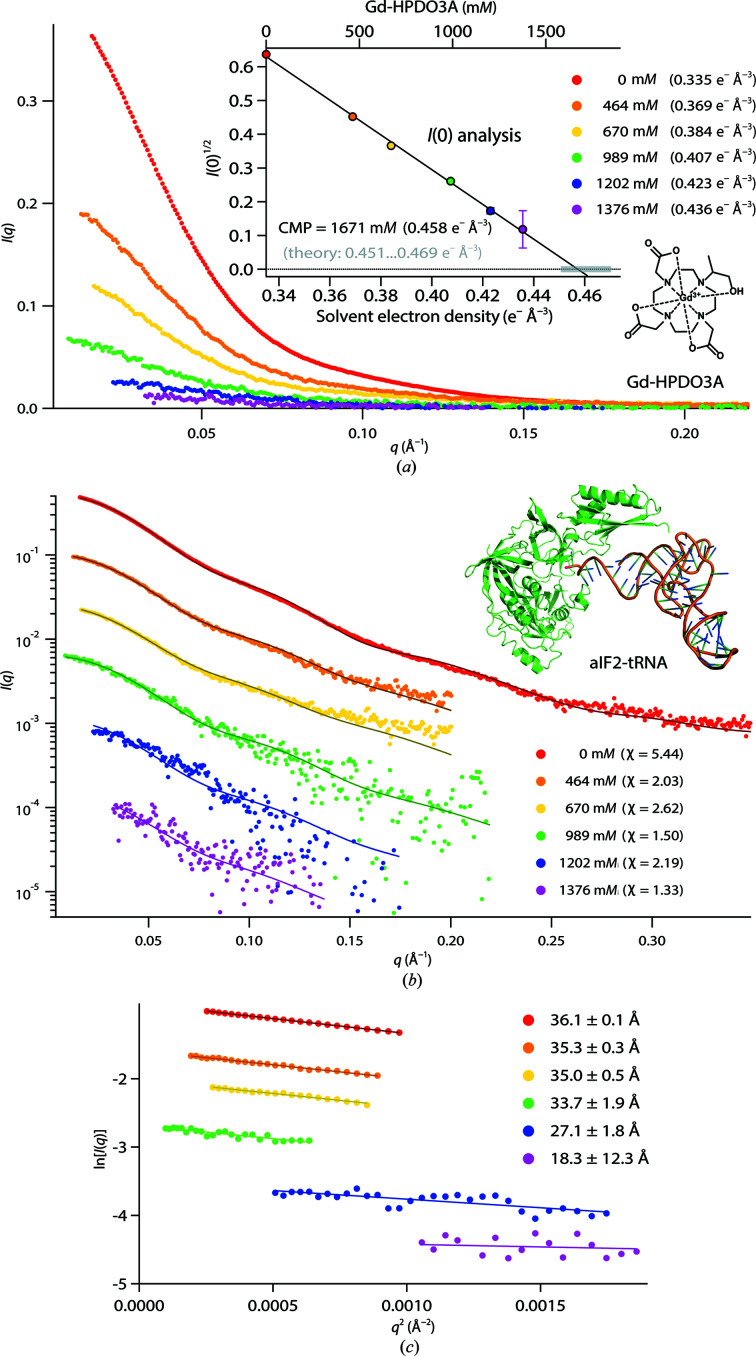
aIF2-tRNA data with Gd-HPDO3A. (*a*) SAXS curves (linear scale) at various Gd-HPDO3A concentrations. Inset: the square root of the scattering intensity at zero angle, *I*(0)^1/2^, and linear fit for determination of the CMP. (*b*) *CRYSOL* fits with an aIF2-tRNA atomic model (constructed from PDB entry 3v11; Schmitt *et al.*, 2012 ▸; see supporting information), logarithmic scale. For reasons of legibility, the SAXS curves were shifted. (*c*) Guinier fits used to determine the *R*_g_ and *I*(0) values.

The aIF2-tRNA complex was measured in Gd-HPDO3A at up to 1376 m*M*, corresponding to a maximum solvent density of 0.436 e^−^ Å^−3^. While the SAXS signal decreased continuously over the concentration range (Fig. 2 ▸ *a*), it did not disappear completely, and an appreciable form factor was present even at the highest Gd-HPDO3A concentration (Fig. 2 ▸ *b*, pink points). Indeed, due to the contribution of the RNA density, the experimentally determined CMP of the complex (0.458 e^−^ Å^−3^) is higher than that of a typical protein and was not reached (Fig. 2 ▸ *a*, inset). The *CRYSOL* fits of the atomic model (constructed from PDB entry 3v11, see supporting information) with the experimental SAXS curves yielded good agreements at almost all solvent electron densities probed, suggesting that the complex maintained its structural and conformational integrity up to the highest Gd-HPDO3A concentrations. Likewise, the strict linearity of *I*(0)^1/2^ (Fig. 2 ▸ *a*, inset) indicates the absence of dissociation or aggregation of aIF2-tRNA over the entire concentration range. Finally, the experimental CMP was within the range of values calculated from the amino-acid and nucleotide volumes of the complex (0.451–0.469 e^−^ Å^−3^; Supplementary Table S1).

Altogether, the aIF2-tRNA SAXS data suggest that Gd-HPDO3A molecules are inert towards this protein–RNA complex and their presence (at up to molar concentrations) neither affects the structural integrity of the complex nor generates any appreciable solvation effects or specific interactions with Gd-HPDO3A molecules.

### 3.2. Iohexol increases solvent electron densities more efficiently than Gd-HPDO3A, but interacts specifically with P1 and aggregates the aIF2-tRNA complex

**Figure 3:**
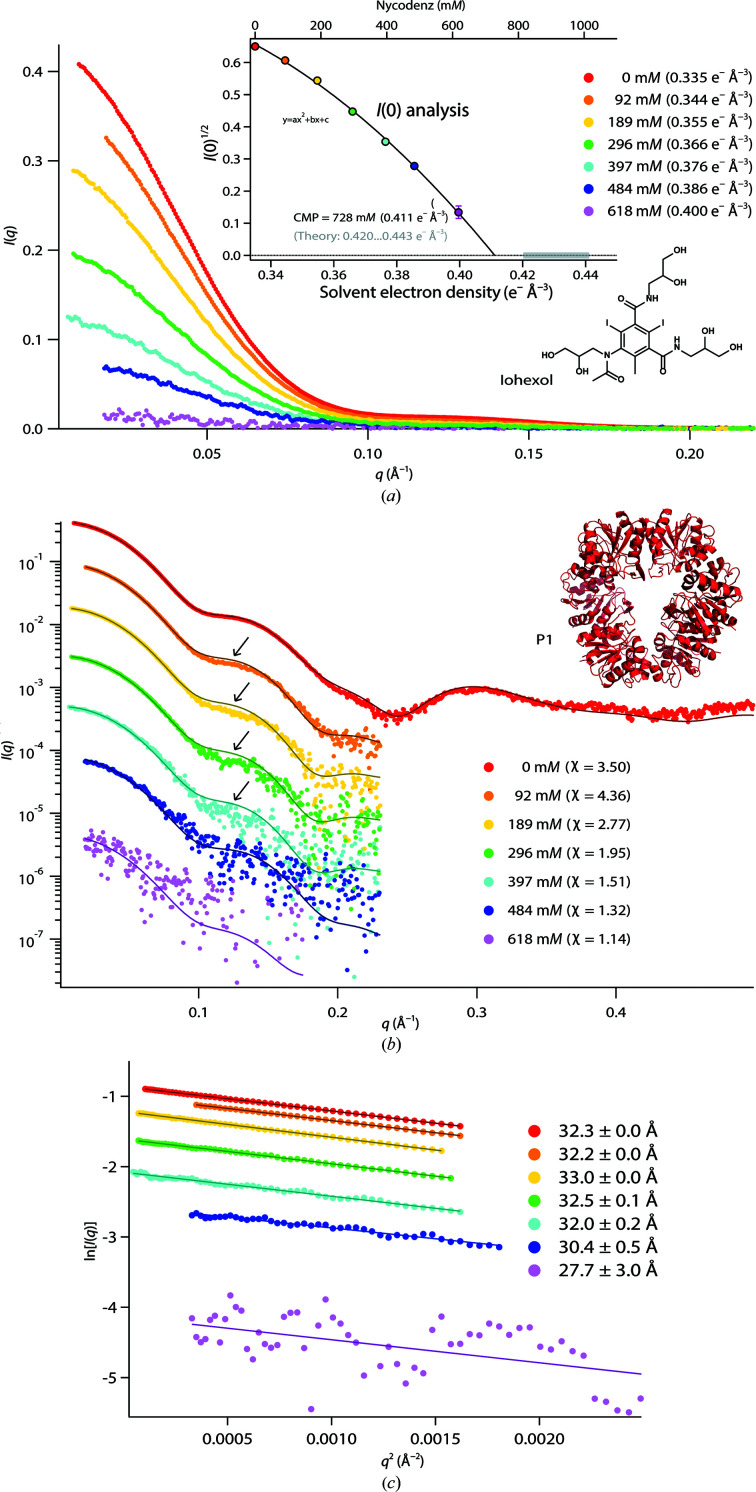
P1 data with iohexol. (*a*) SAXS curves (linear scale) at various iohexol concentrations. Inset: the square root of the scattering intensity at zero angle, *I*(0)^1/2^, and quadratic fit (continuous line) for determination of the CMP. (*b*) *CRYSOL* fits with a hexameric P1 model (PDB entry 7qo8; see supporting information), logarithmic scale. For reasons of legibility, the different SAXS curves were shifted. Arrows indicate *q*-ranges with systematic deviations between the theoretical and experimental data. (*c*) Guinier fits used to determine the *R*_g_ and *I*(0) values.

P1 was measured in iohexol up to 618 m*M*, yielding a maximum solvent electron density of 0.400 e^−^ Å^−3^ (Fig. 3 ▸ *a*). While this value was inferior to the maximum value obtained with Gd-HPDO3A, iohexol is more efficient (per mole) in increasing solvent electron densities (Supplementary Table S2). The hexameric state and overall conformation of P1 remained intact up to the highest concentration, as revealed by *CRYSOL* fits with the atomic model (PDB entry 7qo8; Fig. 3 ▸ *b*) and stable *R*_g_ values (Fig. 3 ▸ *c*). However, the fits of atomic P1 models in the presence of iohexol displayed a systematic and significant mismatch at a shoulder of the SAXS data between 0.1 and 0.15 Å^−1^ (Fig. 3 ▸ *b*, arrows) and a plot of *I*(0)^1/2^ did not display the linear behavior expected for an inert contrast agent (Fig. 3 ▸ *a*, inset). Rather, it was necessary to apply a phenomenological quadratic equation in order to obtain a satisfactory agreement with the forward-scattered intensities. The CMP thus determined (0.411 e^−^ Å^−3^) was significantly lower than the range of values expected from the protein sequence (0.420–0.443 e^−^ Å^−3^). Together, our data suggest that while preserving the oligomeric state of P1, iohexol preferentially interacts with the protein hexamer and/or changes the solvent properties close to its surface.

In order to probe whether the specific binding of iohexol molecules can improve the quality of the SAXS fits, we generated P1 models with a single iohexol molecule bound in two distinct, opposite locations: either outside or inside the hexameric ring (Supplementary Fig. S1). P1 structures with an iohexol molecule modeled inside the ring improved the *CRYSOL* fit significantly, in particular in the shoulder region between 0.1 and 0.15 Å^−1^ (92 m*M* iohexol data; Supplementary Fig. S1*a* ), while the model with an iohexol molecule placed outside the ring decreased the quality of the fit. Importantly, the fits of the 0 m*M* data (negative control; Supplementary Fig. S1*b* ) deteriorated in the presence of an iohexol molecule placed at either location. Fits of all iohexol SAXS data sets, placing a variable number of molecules inside the P1 ring, revealed a tendency to increased specific binding at higher solvent concentrations but reaching a saturation of about three bound iohexol molecules at intermediate concentrations (Supplementary Fig. S2).

**Figure 4:**
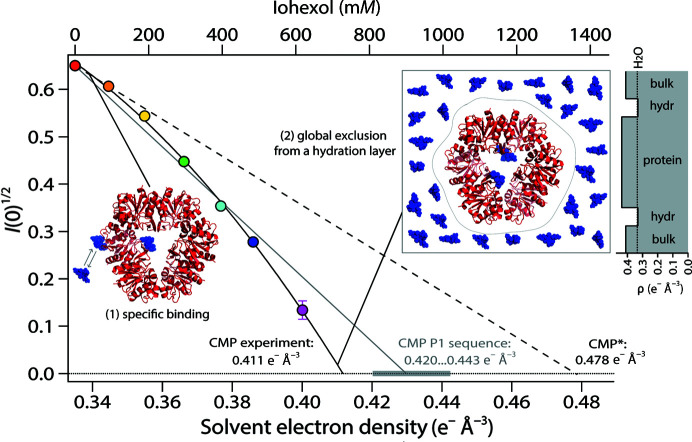
P1 contrast analysis in the presence of iohexol. Square roots of the forward-scattered intensities by P1, *I*(0)^1/2^, measured at various iohexol concentrations are indicated by colored circles and are identical to those shown in the inset in Fig. 3 ▸(*a*). The experimental CMP is extrapolated from a phenomenological quadratic fit and is below that expected for P1 from its sequence (shaded gray area). *I*(0)^1/2^ does not display the linear behavior expected for an isolated protein (continuous gray line). The slope at the origin (linear fit through red and orange data points, broken black line) would yield an ‘apparent’ contrast-match point (CMP*) well above that expected from the P1 sequence. We explain the complex behavior of *I*(0)^1/2^ by two concomitant phenomena: (1) specific binding of 2–3 iohexol molecules to the P1 surface at low concentrations (and reaching saturation at intermediate concentrations) and (2) global exclusion of iohexol molecules from a hydration layer/shell around the P1 surface, in particular at higher concentrations.

Alternatively and independently, the number of bound iohexol molecules at the lowest concentration (92 m*M*, 0.344 e^−^ Å^−3^) was quantified in a model-free approach *via* the slope of the forward-scattered intensities at the origin (Fig. 4 ▸, broken black line): assuming the binding of *N* iohexol molecules per P1 hexamer at 92 m*M*, the relative change of *I*(0)^1/2^ at the origin can be calculated from the respective volumes and contrasts of P1 and iohexol (equation S2). Applying the values in Supplementary Tables S1 and S3 yielded an average of ∼2.4 iohexol molecules bound per P1 hexamer at 92 m*M*. This number is in excellent agreement with *CRYSOL* fits of atomic models against the entire SAXS curve, showing that lowest χ values are obtained when two iohexol molecules are bound at the inside of the P1 ring (Supplementary Fig. S2*b* , inset).

While the binding of ∼2–3 iohexol molecules at low concentrations describes the *I*(0)^1/2^ slope at the origin accurately, a further increase in binding with concentration would lead to a CMP* of 0.478 e^−^ Å^−3^ (Fig. 4 ▸, broken black line), which is significantly higher than the experimentally observed CMP. Surprisingly, the experimental CMP is below even that predicted from the protein sequence (Fig. 4 ▸, area shaded in gray), *i.e.* without bound iohexol molecules. A potential mechanism capable of decreasing the CMP at high iohexol concentrations would be a P1 hydration shell with lower average electron densities than the bulk (equation S3). Using the values in Supplementary Tables S1 and S2, the condition *I*(0)^1/2^ = 0 yields a P1 hydration-shell contrast Δρ_hydr_ of −0.08 e^−^ Å^−3^ at the CMP, *i.e.* 20% less dense than the bulk (ρ_bulk_ = 0.411 e^−^ Å^−3^), or an absolute value ρ_hydr_ = 0.331 e^−^ Å^−3^, which is close to that of an aqueous solution (Fig. 4 ▸, top right inset). This value would require a very strong diminution of the local iohexol concentration in this shell, *i.e.* would correspond to the global exclusion of iohexol molecules (apart from those already specifically bound to P1; Fig. 4 ▸, top right inset).

In order to corroborate the presence of a hydration shell of lower density than the bulk, we fitted the hydration-shell densities of P1 atomic models with two iohexol molecules bound at the inside (Supplementary Fig. S2*h* ) with *CRYSOL* against the experimental SAXS data. Complementarily, we imposed fixed values of hydration-shell densities in *CRYSOL*, calculated the corresponding theoretical SAXS curves of the models and scored the resulting theoretical *R*_g_ against the experimental values. The results from both approaches are shown in Supplementary Fig. S3 and yielded consistent values. It should be noted that ‘standard’ *CRYSOL* fits (*i.e.* using the hydration-shell density as a fit parameter) are limited to positive values and yielded zero at the highest iohexol concentrations. The screening of theoretical SAXS curves with imposed fixed hydration-shell densities, on the other hand, revealed that negative values for the hydration shell do indeed yield the best agreements between the experimental and the theoretically predicted *R*_g_ at high iohexol concentrations and thus corroborate the *I*(0) analysis.

The aIF2-tRNA complex was measured in iohexol at 307 and 604 m*M* (Supplementary Fig. S4). Unfortunately, at both concentrations the very strong increase (of several orders of magnitude) in the scattered intensities at low *q*-values indicated the presence of large and nonspecific aggregates. Thus, iohexol, even at moderate concentrations, destabilizes the structural integrity of this protein–RNA complex and leads to unfavorable interactions promoting aggregation. A sophisticated structural analysis was therefore not attempted.

### 3.3. Interaction and structural properties of free contrast agents

**Figure 5:**
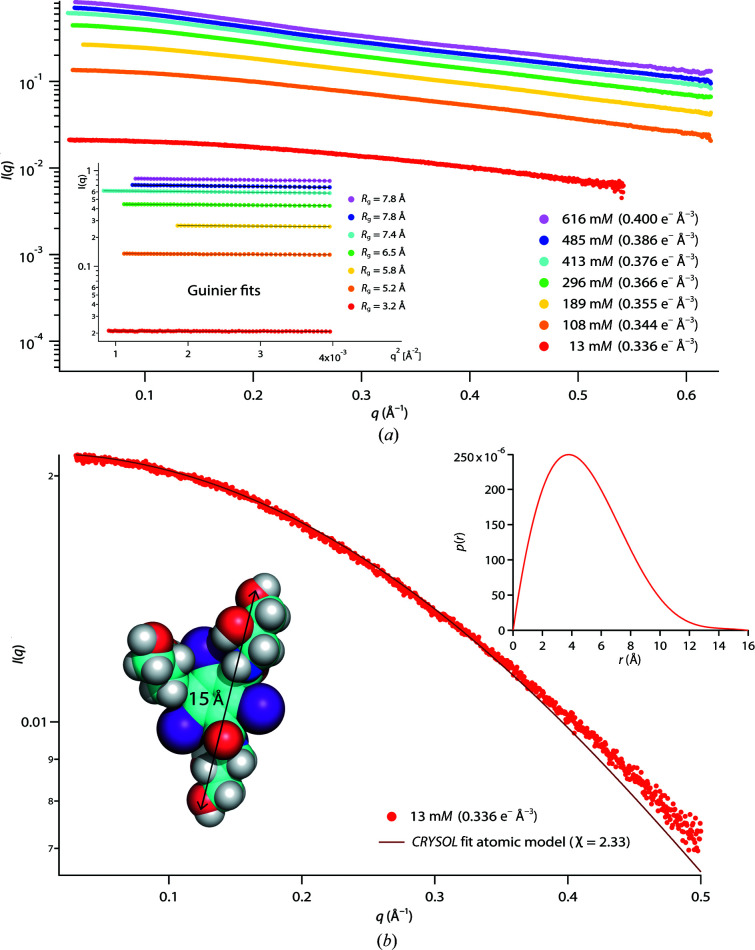
SAXS data for free iohexol. (*a*) SAXS curves at variable iohexol concentrations. No scaling was applied. Inset: Guinier fits and extracted *R*_g_ using *PRIMUS*. (*b*) Iohexol SAXS curve at the lowest measured concentration (13 m*M*). Left bottom inset: iohexol molecule (PubChem) and the distance between two distal O atoms (*PyMOL*). The continuous fit to the SAXS data was carried out with *CRYSOL* from the atomic model. Top right inset: pair distance distribution function *p*(*r*) (arbitrary units), extracted with *GNOM*.

The SAXS data recorded allow the structures of P1 and aIF2-tRNA to be analyzed and their interactions with the contrast agents to be characterized, but at the same time encode information on the structures and interactions of the isolated, *i.e.* free, contrast agents themselves. In order to extract this information, we subtracted the respective aqueous buffers (0 m*M*) from the buffer solutions containing iohexol or Gd-HPDO3A (but no biomacromolecules). Both contrast agents display a qualitatively very distinct behavior (Figs. 5 ▸ and 6 ▸).

Iohexol displays weak attractive inter-particle interactions, resulting in increasing *R*_g_ values with concentration (Fig. 5 ▸ *a*, inset), *i.e.* iohexol molecules have a tendency to oligomerize at higher concentrations. An analysis of the SAXS curve at the lowest concentration (13 m*M*) yielded an *R*_g_ of 3.2 Å and a maximum dimension *D*_max_ of ∼16 Å (Fig. 5 ▸ *b*, top right inset), in good agreement with the distance between the most distal O atoms (∼15 Å) of the atomic model (Fig. 5 ▸ *b*, bottom left inset). The asymmetry of the *p*(*r*) function, with a maximum situated at a relatively short distance (∼4 Å) with respect to its *D*_max_ (∼16 Å), reflects the concentration of high electron densities close to the center region of the molecule due to the presence of three I atoms in the inner ring. Finally, a *CRYSOL* fit of the atomic iohexol model (https://pubchem.ncbi.nlm.nih.gov/compound/Iohexol) yielded very good agreement with the experimental data at 13 m*M* (Fig. 5 ▸ *b*). In conclusion, the SAXS data revealed that free iohexol molecules display weak attractive inter-particle interactions: they are predominantly in a monomeric form at low (∼10 m*M*) concentrations, in good agreement with the atomic model, but have a tendency to associate into small oligomers at higher concentrations.

**Figure 6:**
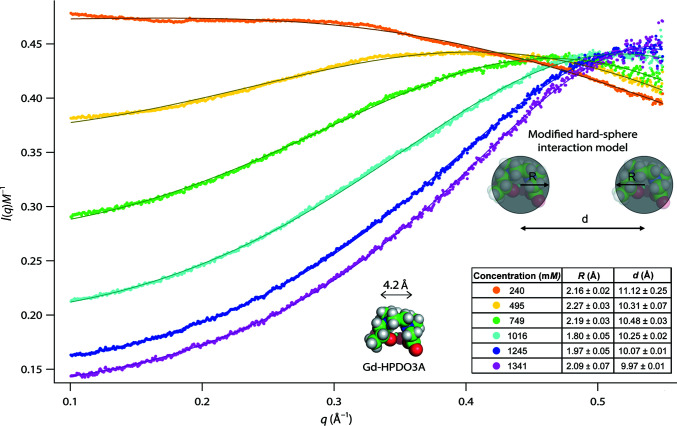
SAXS data for free Gd-HPDO3A. SAXS curves at variable concentrations, normalized against concentration. The continuous lines represent fits with a modified hard-sphere model (equation S4): Gd-HPDO3A molecules are represented by homogeneous spheres of radius *R* that move freely in solution but cannot approach each other closer than a distance *d* between their centers. Insets: table with fit parameters and representations of an atomic Gd-HPDO3A model (*PyMOL*). 4.2 Å corresponds to the distance between opposite N atoms in the macrocycle.

Free Gd-HPDO3A displayed a qualitatively very distinct behavior from iohexol: the decreasing intensities at low angles indicate the presence of strong repulsive inter-particle interactions at higher concentrations (Glatter, 2018 ▸). Analytical expressions can only be given for a limited number of such interaction potentials and under certain assumptions (Lindner & Zemb, 2002 ▸; Guinier & Fournet, 1955 ▸). Based on the fact that Gd-HPDO3A molecules carry no net charge, we applied a classical hard-sphere interaction potential to describe their interaction (equation S4), but failed to fit the experimental data in a satisfactory manner (Supplementary Fig. S5). Only when the interaction distance *d* between the spheres was relaxed and allowed to adopt values greater than twice the sphere radius *R* was it possible to fit SAXS curves at all concentrations in a satisfactory way (Fig. 6 ▸). Importantly, the fits of this modified hard-sphere potential yielded relatively stable and consistent values of *R* and *d* for all Gd-HPDO3A concentrations (Fig. 6 ▸, inset table), thus corroborating the appropriateness of the model. The radius *R* of 1.8–2.3 Å corresponds to that of a sphere of homogeneous electron density with a diameter of 3.6–4.6 Å. This value is in good agreement with the dimensions of the Gd-HPDO3A molecule (Fig. 6 ▸, bottom inset), considering the fact that most of the electron density (notably the Gd^3+^ ion) is concentrated in its inner part.

Interestingly, the SAXS fits indicate that even though they do not carry a net charge, Gd-HPDO3A molecules cannot approach each other beyond about 10 Å (center-to-center distance). This surprising result could be related to the presence of structural water molecules, similar to those detected to be intercalated between pairs of Gd-HPDO3A molecules in hen egg-white lysozyme crystal structures [PDB entries 1h87 (Girard *et al.*, 2002 ▸) and 4tws (Holton *et al.*, 2014 ▸)]. Indeed, the presence of these structural water molecules imposes a minimum distance of about 6.1–6.5 Å between the Gd^3+^ ions (Supplementary Fig. S5).

## 4. Discussion and conclusions

### 4.1. SAXS can reveal and characterize specific interactions of contrast agents with biomacromolecules

While iohexol was more efficient (per mole) than Gd-HPDO3A in increasing solvent electron densities (Supplementary Table S2), the evolution of the SAXS data revealed that it interacted specifically with P1 (Fig. 4 ▸) and led to aggregation of the aIF2-tRNA complex (Supplementary Fig. S4). Importantly, the quality of the SAXS data was good enough to propose an interaction model of iohexol molecules with the P1 surface, indicating both an approximate location (towards the inner part of the hexameric ring; Supplementary Fig. S1) and the evolution of their number as a function of concentration (Supplementary Fig. S2). Best fits of atomic models with the entire SAXS curve showed that at 92 m*M* about two iohexol molecules were bound per P1 hexamer. This number had a tendency to increase to 3–4 at intermediate concentrations and then reach saturation (Supplementary Fig. S2, insets). The presence and the number of bound iohexol molecules was confirmed independently by a complementary, model-free analysis of the relative changes of the forward-scattered intensities *I*(0) (Fig. 4 ▸). Moreover, the experimental value of the contrast-match point (CMP) revealed that the number of bound iohexol molecules does not increase continuously with concentration over the whole range, and that an opposing effect of a decreasing average electron density of the solvent in the proximity of the P1 surface (a hydration layer/shell) occurs concomitantly.

Together, our data indicate that iohexol molecules interact progressively with P1 at specific sites at low concentrations (<100 m*M*) and reach saturation at intermediate concentrations (200–300 m*M*). In parallel, the *average* electron density of the P1 hydration shell decreases by 10–20% with respect to the bulk at the highest (618 m*M*) iohexol concentrations. This process is not revealed by *CRYSOL* ‘standard’ fits, which optimize the hydration shell (and require Δρ_hydr_ > 0), but is revealed by an analysis of the *R*_g_ values from theoretical and experimental curves (Supplementary Fig. S3) and the CMP (Fig. 4 ▸ and equation S3). An ∼20% decrease (−0.08 e^−^ Å^−3^) of the P1 hydration-shell density with respect to the bulk (0.411 e^−^ Å^−3^) would result in a value similar to that of pure water (0.335 e^−^ Å^−3^), *i.e.* equivalent to the total exclusion of iohexol molecules (apart from the specifically bound molecules) in an ∼3 Å thick layer around P1 at the highest concentrations.

A process of specific binding of a small number of iohexol molecules and simultaneous general exclusion (or a diminution of concentration) in a layer near the protein surface may appear surprising. However, it is known that at high concentrations of co-solutes either preferential exclusion of water or increased hydration can be observed in the vicinity of proteins as a function of the chaotropic/kosmotropic character of the co-solutes (Moelbert *et al.*, 2004 ▸; Timasheff, 2002 ▸). Recent SAXS studies have shown that trehalose and glucose (Ajito *et al.*, 2018 ▸), as well as glycerol (Sinibaldi *et al.*, 2007 ▸) are, as kosmotropic agents, preferentially excluded from protein surfaces. It should also be noted that at molar concentrations iohexol molecules occupy about 50%(*v*/*v*) of the bulk solvent (Supplementary Table S2), and thus minor variations in their local concentration can result in rather large variations in the solvent electron density.

It is interesting to note that while interacting specifically with P1 (and potentially also with the aIF2-tRNA complex, leading to its aggregation), iohexol molecules also display an attractive interaction between themselves (Fig. 5 ▸), while Gd-HPDO3A molecules display repulsive interactions between each other (Fig. 6 ▸). Our data cannot establish a direct connection between the two phenomena, but they constitute a wealth of information for future analyses using more sophisticated methods, including explicit solvent simulations (Knight & Hub, 2015 ▸). While challenging (potentially requiring the optimization of existing force fields), such approaches would have the potential to provide hints regarding the possible molecular mechanisms linking the interactions of specific contrast molecules with each other, with water molecules and with biomacromolecular surfaces.

In conclusion, based on the present data, but also on previous results from DDM micelles (Gabel *et al.*, 2019 ▸), iohexol, while very efficient in increasing solvent electron densities per mole, does not seem to be inert towards several classes of biomolecules and thus is probably not suitable as a universal contrast agent for SAXS experiments.

### 4.2. Gd-HPDO3A appears to be an efficient and inert contrast agent for a broad range of biological systems

While Gd-HPDO3A is less efficient (per mole) than iohexol in increasing solvent electron densities (Supplementary Table S2), it is inert towards P1 and aIF2-tRNA; in other words, it leaves their quaternary structures intact (Figs. 1 ▸ *b* and 2 ▸ *b*) and displays no specific interaction with them (Figs. 1 ▸ *a* and 2 ▸ *a*). Together with previous results on DDM micelles (Gabel *et al.*, 2019 ▸), our data suggest that this family of molecules may be compatible with a broad range of biomacromolecular systems (protein–protein and protein–RNA/DNA complexes, as well as detergent-solubilized membrane proteins) and serve as an efficient contrast agent for biological SAXS experiments.

The inertness of Gd-HPDO3A molecules towards the biomacromolecular complexes studied, *i.e.* the absence of a pronounced specific interaction with them or a modification of the solvent electron density in their vicinity, might be correlated with their specific inter-particle interaction (Fig. 6 ▸). Indeed, Gd-HPDO3A molecules display a strong inter-particle repulsion, suggesting the presence of structural water molecules bound to them (as shown in crystal structures; Holton *et al.*, 2014 ▸; Girard *et al.*, 2002 ▸) and thus preventing direct contact between molecules. If structural water molecules are indeed bound to these lanthanide complexes in solution, their presence could equally influence the direct interaction and binding properties with biomacromolecular surfaces.

### 4.3. Future perspectives and challenges of biomacromolecular SAXS using heavy-atom-based complexes

Our present data from an oligomeric protein and a protein–RNA complex, as well as previous data on DDM micelles (Gabel *et al.*, 2019 ▸), suggest that lanthanide-based complexes such as Gd-HPDO3A are promising candidates as efficient SAXS contrast agents for a broad class of biomacromolecules and assemblies. Indeed, an important advantage of this class of molecules with respect to ‘classical’ contrast agents (for example sugar, glycerol and salt) is that their chemical structure can be modified and solvent electron densities do not exclusively depend on concentration as the unique adjustment parameter. Chemical modifications may alter (and ideally improve) their capacity to increase solvent electron density; for example, the mere replacement of the central Gd^3+^ ion (61 e^−^) in the HPDO3A ligand by Lu^3+^ (68 e^−^) or Bi^3+^ (80 e^−^) would increase the electron density of a 1.46 *M* solution from 0.444 to 0.451 or 0.461 e^−^ Å^−3^, respectively (Supplementary Table S2), assuming that the molecular volume and solubility of the resulting complex are not altered. Moreover, cage groups exist in multiple forms (Caravan *et al.*, 1999 ▸), with potentially better maximum solubilities. Assuming a moderate increase in solubility of 15% (*i.e.* 1.68 *M*) of a complex with a similar volume and number of electrons would yield a solvent electron density of 0.455 e^−^ Å^−3^.

These simple estimations demonstrate that solvent electron densities of 0.47–0.48 e^−^Å^−3^ do not appear to be out of reach using a combination of moderate modifications of the physico-chemical properties of such molecules. Such solvent densities are equal to or even beyond the CMPs of protein–RNA/DNA complexes and would give rise to very specific features of the SAXS curves such as apparent negative *R*_g_ and *p*(*r*) functions with areas of opposite sign (Supplementary Fig. S10). In analogy to SANS contrast variation, SAXS curves close to the CMP are rich in information on the internal topology of such complexes and in particular on the distances between partners displaying opposite contrast (Gabel, 2015 ▸). Obviously, modifications of chemical moieties and substitutions of heavy atoms in these molecules will require cross-checks on the compatibility with the structural integrity of biomacromolecular complexes and their potential interactions.

Additional precious information encoded in SAXS data recorded over broad concentration ranges concerns the internal structure and interactions of the contrast agents themselves (Figs. 5 ▸ and 6 ▸). This structural information can be obtained without recording any additional data by the simple subtraction of an aqueous reference buffer from buffers containing variable amounts of contrast agents but no biomacromolecules (thin lines in Supplementary Figs. S7 and S8). Here, we have limited our analysis to the distinction of attractive (iohexol) and repulsive (Gd-HPDO3A) interactions and the extraction of some basic geometric parameters from a simple model fit of the latter (Fig. 6 ▸, inset). However, this kind of data contains a wealth of information that could be exploited and analyzed in more detail using sophisticated approaches, including molecular-dynamics (MD) simulations using explicit solvent (Knight & Hub, 2015 ▸), while allowing the refinement of molecular force fields at the same time. Moreover, the quality and statistics of SAXS contrast data seem to be sufficient to detect and propose specific interactions of small molecules with biomacromolecular surfaces in favorable cases (Supplementary Figs. S1 and S2). Again, MD simulations with explicit solvent would allow these inter­actions to be modelled and understood in greater detail, potentially in combination with other techniques such as crystallography (Holton *et al.*, 2014 ▸; Girard *et al.*, 2002 ▸) and NMR (Madl *et al.*, 2011 ▸).

Some technical parameters could be varied in future SAXS contrast-variation experiments and may have the potential to improve the quality of the data: firstly, the relatively elevated viscosities of molar solutions of contrast agents require the operation of the standard injection systems with specific parameters in order to assure contiguous liquid columns in the capillary. Secondly, the presence of heavy atoms in the compounds leads to a strong reduction in the sample transmission and increased noise at higher concentrations. While useful as a tool for concentration calibration and checking the correct dilutions of stock solutions (Supplementary Fig. S6), they lead to strong absorption and a decrease in the signal to noise at higher concentrations. A reduction of sample thickness as well as an adjustment of the X-ray wavelength (Henke *et al.*, 1993 ▸) has the potential to improve the signal to noise and should be systematically explored.

In conclusion, new classes of modifiable electron-rich molecules have the potential to considerably improve the state of the art of SAXS solvent contrast variation and provide internal information on macromolecular assemblies of partners with different average electron densities. Thus, SAXS contrast experiments could become more complementary to small-angle neutron scattering (SANS) experiments. SANS experiments have the advantage of using a ‘gentler’ form of contrast variation in an aqueous solvent (by H_2_O/D_2_O exchange) and cover the contrast range of relevant biomolecules (proteins, RNA, DNA, detergents, lipids *etc.*) more readily (Zaccai & Jacrot, 1983 ▸). Furthermore, neutrons offer the unique advantage of global and specific macromolecular labeling in the form of deuteration, thus enabling the distinction of several macromolecules belonging to the same class (*i.e.* different proteins) in larger assemblies (Mahieu & Gabel, 2018 ▸; Haertlein *et al.*, 2016 ▸). However, neutron sources are unfortunately less widespread than synchrotrons and X-ray home sources, exposure times are longer, sample amounts are more demanding and access conditions are more stringent. Finally, while it is difficult to conceive a homogeneous modification of the electron densities of macromolecules in SAXS without altering their function, it has been shown that electron-rich labels (for example gold beads or heavy atoms) can be attached in order to provide structural information, in particular distance restraints (Hartl *et al.*, 2018 ▸; Grishaev *et al.*, 2012 ▸).

### 5. Related literature

The following references are cited in the supporting information for this article: Afonine *et al.* (2012 ▸), Appolaire *et al.* (2014 ▸), Du *et al.* (2000 ▸), Dubiez *et al.* (2015 ▸), Emsley *et al.* (2010 ▸), Guinier (1939 ▸), Kabsch (2010 ▸), Kikhney *et al.* (2020 ▸), Svergun *et al.* (1998 ▸), Trewhella *et al.* (2017 ▸) and Winn *et al.* (2011 ▸). 

## Supplementary Material

PDB reference: protease 1, 7qo8

Details of sample preparation and SAXS data reduction/analysis, and Supplementary Figures and Tables. DOI: 10.1107/S2059798322007392/jc5050sup1.pdf
