# Supplementary material for: Medical contrast agents as promising tools for biomacromolecular SAXS experiments
Source: Acta Crystallogr D Struct Biol. 2022 Aug 9;78(Pt 9):1120–30. doi: 10.1107/S2059798322007392 (PMC9435597; doi:10.1107/S2059798322007392)
Supplement: Supplementary file 1 [file d-78-01120-sup1.pdf]

STRUCTURAL
BIOLOGY

Volume 78 (2022)

Supporting information for article:

Medical contrast agents as promising tools for biomacromolecular SAXS experiments

Frank Gabel, Sylvain Engilberge, Emmanuelle Schmitt, Aurélien Thureau, Yves Mechulam, Javier Pérez and Eric Girard

S1. Sample preparation and calculation of electron densities

Iohexol (IUPAC name: N1,N3-bis(2,3-dihydroxypropyl)-5-[N-(2,3-dihydroxypropyl)acetamido]-2,4,6-triiodobenzene-1,3-dicarboxamide) was purchased from Sigma-Aldrich (catalogue number D2158). Gd-HPDO3A was kindly provided by Bracco Imaging S.p.A., Milan, Italy. Protease 1 (P1) from *Pyrococcus horikoshii* was purified following existing protocols (Engilberge *et al.*, 2017) and concentrated to obtain a 8.4 mg/mL stock solution in aqueous buffer (20 mM Tris pH 7.5, 150 mM NaCl). Gd-HPDO3A and iohexol contrast agent stock solutions were prepared by weighing and dissolving in Milli-Q water (Table S2). Appropriate volumes of the P1 stock solution (and/or buffer) were mixed with appropriate amounts of lyophilized (overnight) contrast agent stock solutions to prepare the final concentration series.

aIF2γ-α(domain3) and Met-$tRNA_f^{Met}$(A1-U72) were prepared following existing protocols (Monestier *et al.*, 2017, Schmitt *et al.*, 2012). The complex was then formed by mixing the proteic component (230 µM final concentration) with nucleic acid component (270 µM final concentration) in 10 mM MOPS-NaOH pH 6.7, 200 mM NaCl, 5 mM $MgCl_2$ containing 1 mM GDPNP. The aIF2γ-α(domain3):GDPNP:Met-$tRNA_f^{Met}$(A1-U72) complex (called aIF2-tRNA for simplification) was then purified by molecular sieving on a Superdex 200 HR column (GE Healthcare) and concentrated to 5 mg/mL using Centricon 30 concentrators. The sample was flash-frozen and stored at -80°C before use. Like in the case of P1, final SAXS samples were prepared by mixing appropriate volumes of the protein-RNA complex solution and/or buffer with appropriate amounts of lyophilized Gd-HPDO3A and iohexol stock solutions.

Nominal solvent electron densities (ρ_{sol}) of the concentration series were calculated based on the weighed amounts of Gd-HPDO3A ($C_{17}H_{29}GdN_4O_7$; 558.7 Da; 279 e^-) and iohexol ($C_{19}H_{26}I_3N_3O_9$; 821.1 Da; 392 e^-), and Milli-Q water (H_2O; 18 Da; 10 e^-) added to prepare stock solutions, and their respective volumes (determined by pipetting) (Table S2). Solvent-excluded volumes of iohexol and Gd-HPDO3A and their respective electron densities (Table S1) were equally determined from these values. The electron densities thus calculated were imposed as fixed bulk solvent densities in all atomic fits with CRYSOL in each contrast agent concentration series (see further below).

S2. Generation of macromolecular atomic models

The PDB model used to interpret the aIF2-tRNA SAXS data was constructed from PDB 3V11 (5 Å resolution (Schmitt *et al.*, 2012)) with the following improvements: the structure of the gamma subunit was replaced by the coordinates from PDB 4RD4 (Dubiez *et al.*, 2015) determined at 1.3 Å resolution, except for regions interacting with the tRNA and with the domain 3 of aIF2a that were kept as in 3V11. Junctions were then regularized in Coot. The coordinate file has been deposited in the SASBDB.

Protease 1 from *Pyrococcus horikoshii* (P1) was prepared and purified as described (Du *et al.*, 2000). Crystals of P1 were obtained by the hanging drop technique by mixing one volume of 12 mg/mL protein solution in aqueous buffer (20 mM Tris pH 7.5 and 150 mM NaCl) and one volume of well solution consisting in 100 mM tri-sodium citrate pH 5.6, 200 mM sodium potassium tartrate, 1.9 to 2.4 M ammonium sulphate. Prior to data collection, the sample was cryo-cooled in liquid nitrogen using a cryo-solution consisting in the crystallization condition supplemented with 25% glycerol.

Diffraction data were collected at the European Synchrotron Radiation Facility (ESRF, Grenoble, France) on FIP-BM30A beamline. Diffraction frames were integrated using the program XDS (Kabsch, 2010), and the integrated intensities were scaled and merged with the programs SCALA and TRUNCATE from the CCP4 program suite (Winn *et al.*, 2011). The structure was determined by molecular replacement using PHASER using as search model the PDB file 1G2I. Refinement was performed with PHENIX (Afonine *et al.*, 2012). The model was successively optimized through iterative cycles of refinement and model building in COOT (Emsley *et al.*, 2010). Data and refinement statistics are summarized in Table S4. The atomic coordinates and measured structure factor amplitudes have been deposited in the Protein Data Bank with accession code 7QO8.

S3. SAXS theory, experiments and data reduction

For isotropically oriented and non-interacting particles in solution, the scattered intensity *I*(q) can be written as (Lindner & Zemb, 2002):

$$I(q) \sim \langle \left| \int_V (\rho - \rho_{sol}) e^{i\vec{q}\cdot\vec{r}} dV \right|^2 \rangle \qquad \text{(Eq. S1)}$$

where $q = \frac{4\pi}{\lambda}\sin\theta$ is the modulus of the scattering vector, λ the X-ray wavelength, and 2θ the scattering angle. The brackets indicate an average over all orientations of the solubilized particles. ρ and ρ_{sol} are the electron densities of the particles and of the bulk solvent, respectively. Importantly, the integral runs over the entire volume of each particle, including areas where the solubilized particle (protein, macromolecular assembly) induces a different electron density than the one of the unperturbed bulk solvent. In particular, it can include a hydration shell in the vicinity of the particle surface (Svergun *et al.*, 1998).

All SAXS experiments were carried out in flow mode on the SWING beamline (*https://www.synchrotron-soleil.fr/en/beamlines/swing*) at SOLEIL synchrotron (Saint Aubin, France), using an X-ray energy of 12.00 or 14.00 keV and a sample-detector distance of 1.79 or 2.00 m. For each sample a volume of 40 µL was circulated at 75 µL/min through a thermalized Quartz capillary of 1.5 mm diameter and 10 µm wall thickness, inserted within a vacuum chamber (David & Perez, 2009). Series (typically 5 to 30) of individual 0.5 or 1 s time frames were collected at 15 ºC on an Aviex CCD

detector. The forward transmitted intensity was measured on a silicon diode based sensor embedded in the beamstop. The 2D scattering patterns were reduced into 1D intensities and binned using the Foxtrot software (*https://www.synchrotron-soleil.fr/en/beamlines/swing*), after manually checking for radiation damage and validation of identical transmissions. No radiation damage was observed in the presence of contrast molecules.

Buffer intensities were subtracted from sample intensities using PRIMUS (Franke *et al.*, 2017), after careful calibration against the logarithm of the measured transmissions (which depends linearly on the concentrations, Fig. S6 A-C), following a previously established protocol (Gabel, Engilberge, Pérez*, et al.*, 2019). This protocol aims at minimizing mismatches between the background levels of buffers and samples which can occur in practice due to slight concentration differences of strongly scattering contrast agents in the molar concentration range used here. While the buffer signals do not evolve linearly over the entire concentration- and q-range (see, e.g., Fig. S7), we found that an approximation of a local linear interpolation, based on the most adjacent concentrations, yielded very satisfactory matches between sample and buffer levels over the whole q-range after calibration (Fig. S6, S7, S8). The stability and limits of this approach are illustrated in Figure S11, which shows that the linear interpolation requires a sufficiently dense series of experimental contrast points. Typically, the spread between adjacent concentrations should not exceed about 20% of the maximum concentration, i.e. a minimum of 5-6 equally spaced data points should be available. Figure S12 shows the entire q-ranges of the resulting subtracted data sets up to lowest and highest values measured. The low (and mostly constant) noise level at high q-values illustrates that buffer background levels match the sample background levels in general well, even though occasionally a slight over-subtraction is observed (detectable by zones of missing data points in the logarithmic plots). At very low angles, a slight mis-match of parasitic scattering was observed in some cases and the respective data points were discarded for the analysis in Figures 1-3 (as were data at high angles that contributed essentially noise).

In the case of the protein and protein-RNA complex, the respective volume fractions (v/v %) were very small (< 0.5% in all cases) and thus not taken into account in the subtraction process. The SAXS curves of the free contrast agents (i.e. in the absence of bio-macromolecules) were obtained by subtracting the respective 0 mM reference buffers from the Gd-HPDO3A and iohexol solutions at various concentrations (Fig. S7 and S8). A weighted subtraction, taking the high (up to 40-50%) volume fractions of contrast molecules into account, did not change the shapes of the subtracted curves and the fitted parameters significantly, as illustrated for Gd-HPDO3A (Fig. S13). This is due to the fact that the background levels of aqueous buffers are much lower than those of buffers containing contrast molecules, and are essentially flat in the q-ranges of interest.

S4. SAXS data analysis

Basic parameters (forward scattered intensity $I(0)$, radii of gyration R_G, maximum dimensions D_{max} and pair distance distribution functions $p(r)$) were extracted from the final 1D curves by using the Guinier approximation (Guinier, 1939) with the programs PRIMUS and GNOM from the ATSAS package (Franke *et al.*, 2017) (Table S3). The experimental contrast match points (CMPs, i.e. $I(0)=0$) of P1 and aIF2-tRNA in Gd-HPDO3A were determined by plotting the square root of the forward scattered intensity, $\sqrt{I(0)}$, vs contrast agent concentration (in mM) and applying a linear fit (Jacrot, 1976). The intersections of the linear fits with the abscissa yielded the concentration of the respective contrast agent where both systems are matched (Fig. 1A and 2A, insets). A linear fit of $\sqrt{I(0)}$ was not possible in the case of P1 in iohexol and a phenomenological fit with the next higher polynomial order (i.e. quadratic) function was applied to determine the experimental CMP (Fig. 3A, inset, and Fig. 4).

Ranges of theoretical solvent-excluded volumes and electron densities of P1 (0.420…0.443 $e^-/Å^3$) and aIF2-tRNA (0.451…0.469 $e^-/Å^3$) were calculated from their sequences (Fig. S9) and amino acid and nucleotide volumes reported in literature (Voss & Gerstein, 2005, Kharakoz, 1997, Creighton, 1993, Jacrot, 1976). Solvent-excluded volumes and electron densities of iohexol and Gd-HPDO3A were determined from their measured volumes (Table S2) and chemical formulae. All parameters are reported in Table S1.

The slope at the origin of the $\sqrt{I(0)}$ P1 iohexol data (i.e. the relative decrease of $\sqrt{I(0)}$ from 0 to 92 mM iohexol) was used to determine the number N of bound iohexol molecules at 92 mM in a model-independent way according to the following equation:

$$\sqrt{\frac{I(0)_{P1,\ 92\,mM}}{I(0)_{P1,\ 0\,mM}}} = \frac{\Delta\rho_{P1,\ 92\,mM}\cdot V_{P1} + N\cdot\Delta\rho_{iohexol,\ 92\,mM}\cdot V_{iohexol}}{\Delta\rho_{P1,\ 0\,mM}\cdot V_{P1}} \qquad \text{(Eq. S2)}$$

Here, $\Delta\rho_{P1,\ 0\,mM}$ = 0.438-0.335 $e^-/Å^3$ = 0.103 $e^-/Å^3$ and $\Delta\rho_{P1,\ 92\,mM}$ = 0.438-0.345 $e^-/Å^3$ = 0.093 $e^-/Å^3$ are the electron density contrasts of P1 at 0 and 92 mM iohexol, and V_{P1} = 136,137 $Å^3$ is the solvent-excluded P1 volume, determined from the number of electrons (59,628) and its experimental CMP in Gd-HPDO3A (Fig. 1A, inset). $\Delta\rho_{Iohexol,\ 92\,mM}$ = 0.625-0.345 $e^-/Å^3$ = 0.280 $e^-/Å^3$ and $V_{Iohexol}$ = 627 $Å^3$ are the electron contrast of iohexol and its molecular volume (Table S1). The ratio $\sqrt{I(0)_{P1,\ 92\,mM}} / \sqrt{I(0)_{P1,\ 0\,mM}}$ was determined from the respective $I(0)$ values determined by the Guinier fits (Table S3) and was 0.933. Note that Eq. S2 neglects potential changes of the overall hydration shell density of P1 in the range between 0 and 92 mM iohexol.

The experimentally determined CMP of P1 in iohexol (Fig. 3A, inset) was determined as 0.411 $e^-/Å^3$ and inferior to the range of values calculated from the protein sequence (Table S1). In order to explain this discrepancy, we postulate a negative average hydration shell contrast $\Delta\rho_{hydr}$ at higher iohexol concentrations, while N iohexol molecules can be bound in parallel to P1 (Eq. S2). The condition $\sqrt{I(0)}$ = 0 at the CMP yields the following equation:

$$\Delta\rho_{P1,\ 618\,mM} \cdot V_{P1} + N \cdot \Delta\rho_{iohexol,\ 618\,mM} \cdot V_{iohexol} + \Delta\rho_{hydr,\ 618\,mM} \cdot V_{hydr} = 0 \qquad \text{(Eq. S3)}$$

V_{P1} and $V_{Iohexol}$ are the P1 and iohexol solvent-excluded volumes and are identical to the ones in Eq. S2. Their respective electron density contrasts at the CMP are $\Delta\rho_{P1,\ 618\ mM}$ = 0.438-0.411 $e^-/Å^3$ = 0.027 $e^-/Å^3$ and $\Delta\rho_{iohexol,\ 618\ mM}$ = 0.627-0.411 $e^-/Å^3$ = 0.216 $e^-/Å^3$. The volume of a hydration shell with a fixed thickness of 3 Å was taken from CRYSOL: V_{hydr} = 42,850 $Å^3$.

The strong inter-particle interactions between free Gd-HPDO3A molecules (which carry no net charge) were analyzed by a modified hard-sphere interaction potential (Fournet, 1951):

$$I(q) = C \cdot \frac{\Phi^2(qR)}{1+\frac{8v_0}{v_1}\Phi(qd)} \qquad \text{(Eq. S4)}$$

C is a factor that depends on sample concentration and the number of electrons per particle, $v_0 = (4/3)\cdot\pi\cdot R^3$ is the volume of a spherical particle with radius R, and v_1 the average volume available for each particle in solution. $\Phi(qa)$ is the form factor of a sphere of radius a:

$$\Phi(qa) = 3 \cdot \frac{\sin(qa) - qa\cdot\cos(qa)}{(qa)^3} \qquad \text{(Eq. S5)}$$

In the case of a strict hard-sphere inter-action potential, d has a constant value of $2R$ and $a = R$. However, we did not obtain satisfactory fits when assuming that Gd-HPDO3A particles can contact each other directly (Fig. S5). Rather, it was necessary to introduce a value $d > 2R$ which allowed to fit Gd-HPDO3A curves at all concentrations in a very satisfactory manner (Fig. 6).

Fits of all atomic models (P1, aIF2-tRNA, iohexol) against experimental SAXS curves were carried out with CRYSOL (version 2.8.2), imposing the bulk solvent electron density as a fixed value, calculated as described above from the values reported in Table S2, applying appropriate dilution factors. Default values of all available parameters (number of harmonics, Fibonacci grid, and maximum q) were used, and an optimization by a constant background subtraction was deactivated. Thus, in general, the only free fit parameter of the CRYSOL fits was the hydration shell.

An exception to this procedure are the results shown in Fig. S3: here, the density of the hydration shell was imposed as fixed in each case (screening a range from -0.03 to +0.03 $e^-/Å^3$) to calculate a theoretical curve and no fit against experimental SAXS data was carried out. The value of the imposed hydration shell density yielding the best agreement between the radius of gyration of the resulting theoretical curve with the respective experimental SAXS curve was identified, and then compared to the value of fits with a variable hydration shell density, as described above.

Table S1 P1, aIF2, tRNA, iohexol and Gd-HPDO3A volumes, molecular masses, partial specific volumes and electron densities

The values for proteins and the RNA were calculated from the sequences (Fig. S9) and residue and nucleotide volume ranges reported in literature (Voss & Gerstein, 2005, Kharakoz, 1997, Creighton, 1993, Jacrot, 1976). The values for iohexol and Gd-HPDO3A were calculated from their chemical formula and solvent-excluded volumes determined from the respective stock solutions (Table S2).

**Molecule**	**Volume [Å^3]**	**Molecular mass [Da]**	**Partial specific volume [cm^3/g]**	**Electron density [e$^-$/Å^3]**
P1 (hexamer)	134,694...141,996	111,390	0.728...0.768	0.420…0.443
aIF2	68,225...72,143	55,987	0.734...0.776	0.417…0.440
tRNA	23,161	24,512	0.569	0.557
aIF2-tRNA	91,386…95,304	80,499	0.684…0.713	0.451…0.469
Iohexol	625	821	0.459	0.627
Gd-HPDO3A	464	559	0.500	0.601

Table S2 Parameters of Gd-HPDO3A and iohexol stock solutions

All concentration series were prepared by lyophilizing appropriate amounts of the stock solutions overnight and re-solubilizing them in appropriate volumes of P1 and aIF2-tRNA stock solutions and/or buffers.

**Sample**	**Powder [mg]**	**MW [Da]**	**Number of e$^-$**	**H_2O [µL]**	**V_{final} [µL]**	**C_{final} [M]**	**Density [e$^-$/Å^3]**
**Gd-HPDO3A**	450	558.7	279	325	550	1.46	0.444
**Iohexol**	993	821.1	392	400	855	1.41	0.490

Table S3 R_G and I(0) values of P1 and aIF2-RNA in Gd-HPDO3A, of P1 in iohexol, and of free iohexol

Guinier fit limits denote the ranges between $R_G \cdot q_{min}$ and $R_G \cdot q_{max}$. These ranges varied slightly within each series, depending on parasitic noise at very small angles and the quality of buffer subtraction at higher angles of each individual data set.

**P1, Gd-HPDO3A**			
Concentration [mM]	R_G [Å]	Guinier fit limits	$I(0)$
0	32.0 ± 0.0	0.218…1.29	0.781 ± 0.000
234	32.6 ± 0.1	0.425…1.29	0.539 ± 0.001
507	31.8 ± 0.2	0.433…1.28	0.336 ± 0.001
730	32.9 ± 0.6	0.709…1.31	0.169 ± 0.001
1016	32.4 ± 1.1	0.708…1.32	0.048 ± 0.001
1245	32.5 ± 15.8	0.643…1.25	0.008 ± 0.003
1341	25.0 ± 81.1	0.305…1.00	0.007 ± 0.006

**aIF2-RNA, Gd-HPDO3A**			
Concentration [mM]	R_G [Å]	Guinier fit limits	$I(0)$
0	36.1 ± 0.1	0.575…1.06	0.405 ± 0.000
464	35.3 ± 0.3	0.492…1.05	0.204 ± 0.001
670	35.0 ± 0.5	0.580…1.02	0.134 ± 0.001
989	33.7 ± 1.9	0.335…0.85	0.068 ± 0.001
1202	27.1 ± 1.8	0.610…1.13	0.030 ± 0.001
1376	18.3 ± 12.3	0.596…0.79	0.014 ± 0.003

**P1, iohexol**			
Concentration [mM]	R_G [Å]	Guinier fit limits	$I(0)$
0	32.3 ± 0.0	0.347…1.30	0.423 ± 0.000
92	32.2 ± 0.0	0.602…1.30	0.368 ± 0.000
189	33.0 ± 0.1	0.281…1.29	0.296 ± 0.000
296	32.5 ± 0.1	0.276…1.29	0.200 ± 0.000
397	32.0 ± 0.2	0.218…1.29	0.125 ± 0.000
484	30.4 ± 0.5	0.551…1.29	0.077 ± 0.001
618	27.7 ± 3.1	0.501…1.39	0.018 ± 0.001

**Free iohexol**			
Concentration [mM]	R_G [Å]	Guinier fit limits	$I(0)$
13	3.20 ± 0.40	0.096…0.200	0.021 ± 0.000
108	5.16 ± 0.10	0.172…0.324	0.137 ± 0.000
189	5.76 ± 0.07	0.248…0.362	0.272 ± 0.000
296	6.49 ± 0.04	0.217…0.408	0.453 ± 0.000
413	7.40 ± 0.03	0.214…0.465	0.625 ± 0.000
485	7.81 ± 0.04	0.275…0.491	0.725 ± 0.000
616	7.81 ± 0.07	0.279…0.491	0.846 ± 0.001

Table S4 X-ray data collection, processing and refinement statistics for P1.

Data Collection	
**Light source**	ESRF, FIP BM30A
**Wavelength (Å)**	0.9797
**Space group**	$P2_12_12_1$
**Cell constants**	a = 115.74, b = 123.63, c = 129.22 Å
**Resolution (Å)**	70.72-1.95 (2.05-1.95)
**# reflections**	895815 (124000)
**# unique reflections**	135285 (19324)
**Multiplicity**	6.6 (6.4)
**I/σ (I)**	11.80 (1.60)
**Completeness (%)**	99.80 (98.60)
R_{meas}[b] **(%)**	17.8 (173.8)
R_{pim}[c] **(%)**	6.8 (67.3)
$CC_{1/2}$	99.7 (56.7)
Refinement	
R_{work}	0.181
R_{free}	0.203
**rmsd bonds (Å)**	0.010
**rmsd angles (°)**	1.16
**# Number of non-h atoms**	
**Protein**	7862
**Ligands**	40
**water**	1013
**Ave. B-factor (Å²)**	30.90
**Clashscore**	2.66
**Ramachandran analysis,**[d] **% residues in**	
**favoured regions**	98.88
**allowed regions**	1.12
**PDB code**	7QO8

[a]Values in parentheses correspond to the highest resolution shell [b]$R_{meas} = \sum_{hkl} \sqrt{(n/n-1)}\sum_I |I_i(hkl) - \langle I(hkl)\rangle| / \sum_{hkl}\sum_i I_i(hkl)$; [c]$R_{pim} = \sum_{hkl} \sqrt{(1/n-1)}\sum^{n}_{i=1} |I_i(hkl) - \langle I(hkl)\rangle| / \sum_{hkl}\sum_i I_i(hkl)$; [d]Calculated in MolProbity.

Table S5 Essentials on SAS data acquisition, sample details, data analysis, modelling fitting and software used (Trewhella *et al.*, 2017).

(*a*) Sample details

	P1 in iohexol	P1 in Gd-HPDO3A	aIF2-tRNA in iohexol	aIF2-tRNA in iohexol
Organism	*Pyrococcus horikoshii*	*Pyrococcus horikoshii*	*Saccharolobus solfataricus*	*Saccharolobus solfataricus*
Source (Catalogue No. or reference)	-	-	-	-
Description: sequence (including Uniprot ID + uncleaved tags), bound ligands/modifications, *etc.*	Table S9	Table S9	Table S9	Table S9
Extinction coefficient ε at 0.1% and 280 nm	13.66	13.66	9.83[a]	9.83[a]
Partial specific volume $\bar{\upsilon}$ ($cm^3\,g^{-1}$)	0.728…0.768	0.728…0.768	0.684…0.713	0.684…0.713
Mean solute and solvent scattering length densities and mean scattering contrast $\Delta\bar{\rho}$ (cm^{-2})	Table S1, S2	Table S1, S2	Table S1, S2	Table S1, S2
Molecular mass *M* from chemical composition (Da)	111,390	111,390	80,499	80,499
For SEC-SAS, loading volume/concentration, (mg/ml) injection volume (μl), flow rate (ml/min)	-	-	-	-
Concentration (range/values) measured and method[b]	8.4 mg/mL	8.4 mg/mL	5.0 mg/mL	5.0 mg/mL
Solvent composition and source	See M&M	See M&M	See M&M	See M&M

(*b*) SAS data collection parameters

Source, instrument and description or reference:
SWING/SOLEIL U20 in-vacuum undulator
Wavelength (Å):
1.03 or 0.89
Beam geometry (size, sample-to-detector distance):
570x200 μm^2 (H x V), 1.79 or 2.00 m
q-measurement range ($Å^{-1}$):
0.001…0.62[c]
Absolute scaling method:
Water measurement
Basis for normalization to constant counts:
Active beamstop: diamond-based diode
Method for monitoring radiation damage, X-ray dose where relevant:
Comparison of individual frames
Exposure time, number of exposures:
0.5 or 1s per frame. Typically 5-30 frames binned.
Sample configuration including path length and flow rate where relevant:
Glass capillary 1.5 mm. Flow rate 75 µL/min.
Sample temperature (°C):
15

(*c*) Software employed for SAS data reduction, analysis and interpretation

SAS data reduction to sample–solvent scattering, and extrapolation, merging, desmearing *etc.* as relevant:

Foxtrot (*https://www.synchrotron-soleil.fr/en/beamlines/swing*), **ATSAS** **(version 2.8.2)** (Franke *et al.*, 2017)

Calculation of ε from sequence:

-

Calculation of $\Delta\bar{\rho}$ and $\bar{\upsilon}$ values from chemical composition:

Using Excel tables, based on information provided in Jacrot (Jacrot, 1976), **Creighton** (Creighton, 1993), **Kharakoz** (Kharakoz, 1997) **and Voss & Gerstein** (Voss & Gerstein, 2005).

Basic analyses: Guinier, *P*(*r*), scattering particle volume (*e.g.* Porod volume V_P or volume of correlation V_c):

ATSAS **(version 2.8.2)** (Franke *et al.*, 2017)

Shape/bead modelling:

-

Atomic structure modelling (homology, rigid body, ensemble):

From crystallography. See details from SI material.

Modelling of missing sequence from PDB files:

-

Molecular graphics:

PyMOL

(*d*) Structural parameters

Guinier Analysis	P1 in iohexol	P1 in Gd-HPDO3A	aIF2-tRNA in iohexol	aIF2-tRNA in Gd-HPDO3A
$I(0)$ (cm^{-1})	Table S3			
R_g (Å)				
q-range (Å^{-1})				
Quality-of-fit parameter (with definition)	-	-	-	-
M from $I(0)$ (ratio to expected value)	-	-	-	-
$P(r)$ analysis	**13 mM iohexol (Fig. 5)**			**Synthetic data (Fig. S10)**
$I(0)$ (cm^{-1})	-			-
R_g (Å)	3.9			-56[d]
d_{max} (Å)	16	-	-	110
q-range (Å^{-1})	0.03…0.39			0.00…0.50
Quality-of-fit parameter (with definition)	-			-
M from $I(0)$ (ratio to expected value)	-			-
Volume (*e.g.* V_P and/or V_c)	-			-

(*e*) Shape modelling results (a complete panel for each method)

q-range for fitting	Not applied
Symmetry/anisotropy assumptions	
Ambiguity measure(s) with definitions	
χ^2 value/range	
P value, any other quality-of-fit parameters	
Adjustable parameters in the model fit	
Model volume and/or M estimate	
Model precision/resolution	
For multiple phase shape models, R_g values and relative phase volumes	

(*f*) Atomistic modelling

	P1 in iohexol	P1 in Gd-HPDO3A	aIF2-tRNA in Gd-HPDO3A
Method	CRYSOL (Svergun *et al.*, 1995)		
q-range for fitting	Figure 3B, S1, S2	Figure 1B	Figure 2B
Symmetry assumptions	-	-	-
Any measures of model precision	-	-	-
χ^2 value/range	Figure 3B, S1, S2	Figure 1B	Figure 1B
P value, any other quality-of-fit parameters	-	-	-
Adjustable parameters in the model fit	Number and position of bound iohexol molecules. Hydration shell.	Hydration shell.	Hydration shell.
Relevant output parameters (*e.g.* predicted R_g/d_{max} values, weights for multi-state models, *etc.*)	-	-	-

Domain/subunit coordinates and contacts, regions of presumed flexibility as appropriate	-	-	-
***(g)* Data and model deposition IDs**			
	P1 in iohexol	P1 in Gd-HPDO3A	aIF2-tRNA in Gd-HPDO3A
	7QO8 and SI	7QO8	3V11 and SI

[a]Protein alone.
[b]Concentrations of stock solutions, measured by OD at 280 nm (proteins), and 260 nm (RNA). Final sample concentrations depended on volume mixtures indicated in Table S2.
[c]Maximum q-range were data point were available. Final datasets are more restricted due to the elimination of parasitic scattering at very low angles and due to noise at high angles.
[d]R_G is actually a complex number since R_G^2 is negative.

S5.

Additional SI material

The SAXS curves and the CRYSOL fit of the atomic macromolecular structures from Figures 1 to 3 have been deposited in the SASBDB databank (Kikhney *et al.*, 2020) and can be accessed and downloaded using the following links:

https://www.sasbdb.org/data/SASDNM7/ogcrwv7vk2

https://www.sasbdb.org/data/SASDNN7/ynugsvp705

https://www.sasbdb.org/data/SASDNP7/7vxd4b1s1d

aIF2-tRNA and P1 atomic models, as well as P1 structures with iohexol molecules are submitted as additional SI material.

SAXS curves of free contrast agents iohexol and Gd-HPDO3A are available from the authors upon request.

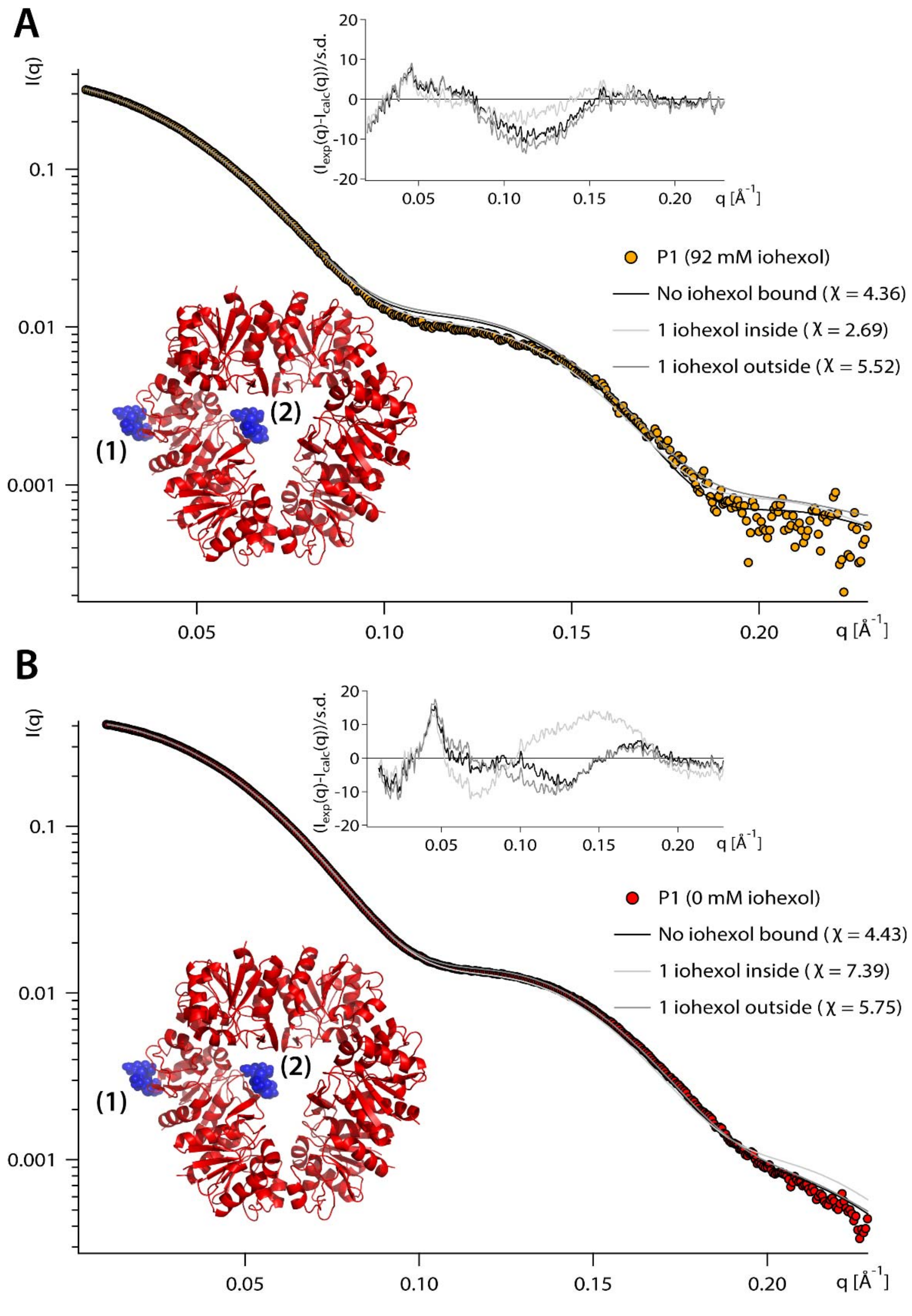

Figure S1 Influence of bound iohexol molecules on fits of SAXS curves. (A) CRYSOL fits and χ values of P1 in 92 mM iohexol: in the absence of bound iohexol molecules (black line), with a single iohexol molecule bound outside ((1), dark gray line) or inside ((2), light gray line) of the hexameric ring. *Inset*: normalized residuals (experimental minus calculated intensities, divided by standard deviation of the experimental errors). (B) CRYSOL fits and χ values of P1 in 0 mM iohexol: in the absence of bound iohexol molecules (black line), with a single iohexol molecule bound outside ((1), dark gray line) or inside ((2), light gray line) of the hexameric ring. *Inset*: normalized residuals.

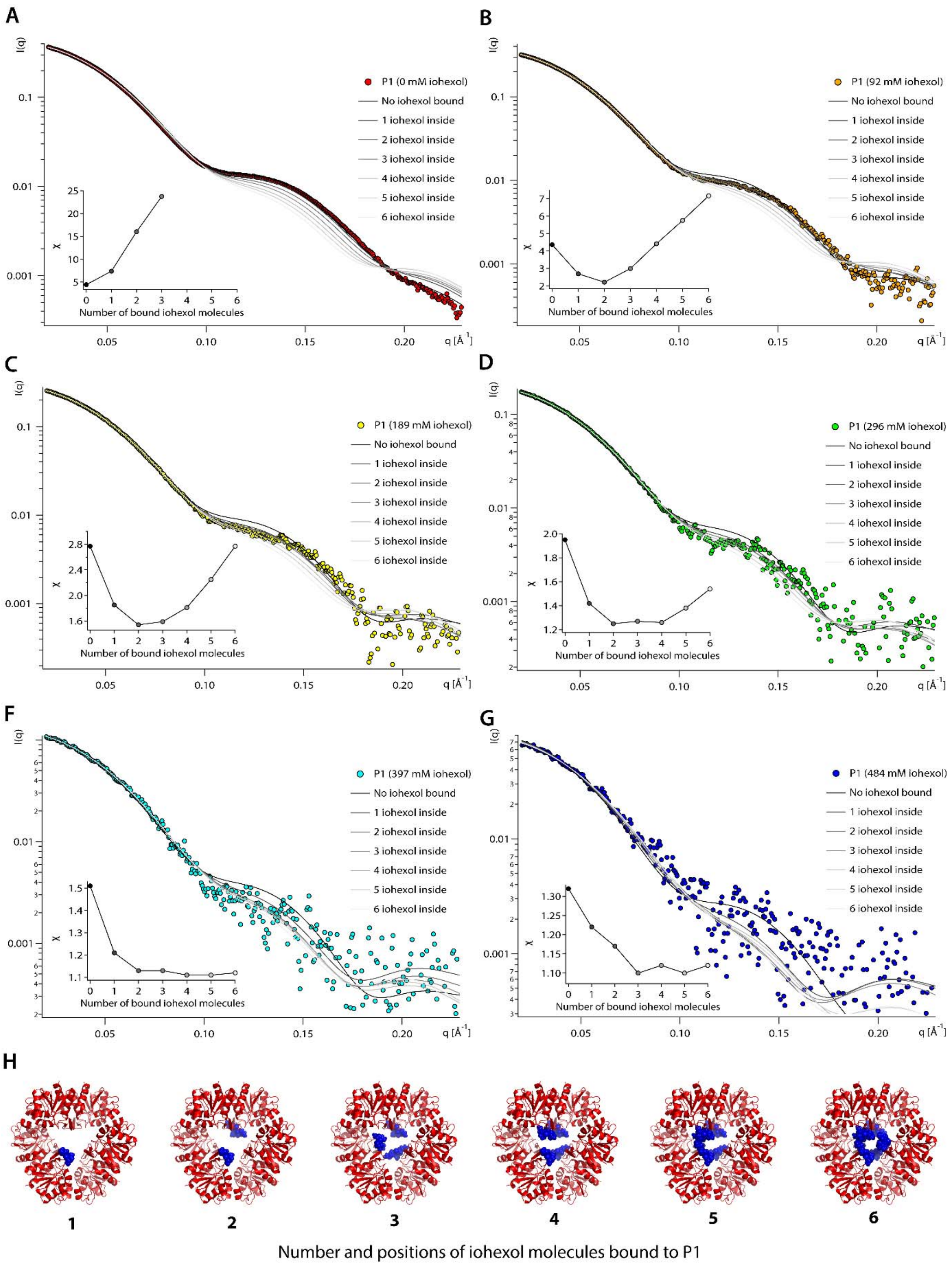

Figure S2 CRYSOL fits as a function of the number of iohexol molecules bound to P1. (A-G) Fits of P1 models, including variable numbers of bound molecules, to experimental SAXS data at 0, 92, 189, 296, 397 and 484 mM iohexol. *Insets*: Respective χ values as a function of the number of iohexol molecules bound. (H) P1 models generated with 1, 2, 3, 4, 5 or 6 iohexol molecules bound at the inside of the hexameric ring, and used for the fits shown in (A-G).

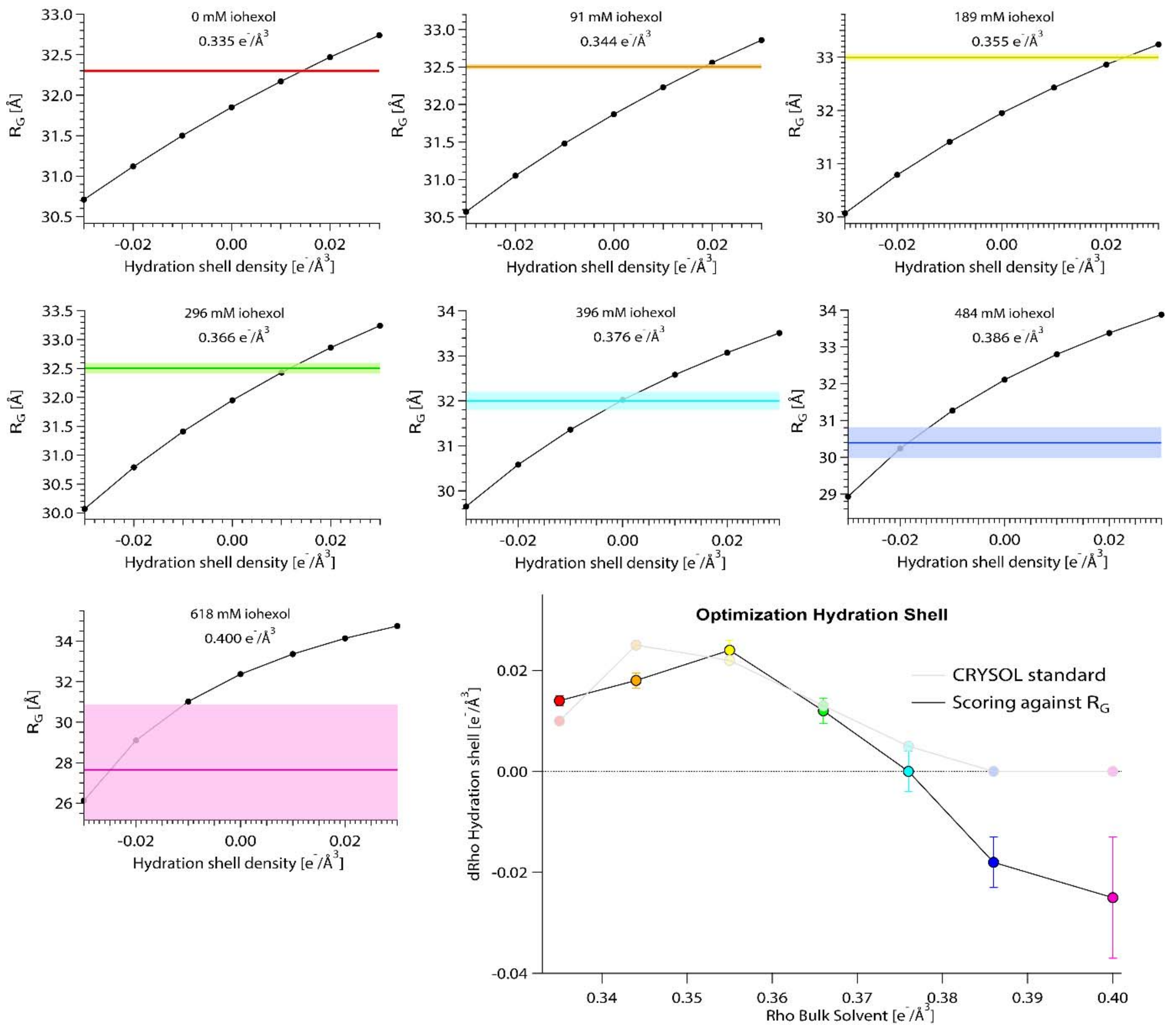

Figure S3 P1 hydration shell contrast in iohexol. *Top*: comparison of experimental radii of gyration (R_G, colored lines including error bars, Fig. 3C) at different iohexol concentrations with the ones extracted from theoretical CRYSOL curves from P1 models (including two fixed iohexol molecules) with imposed, fixed values of the hydration shell densities (black dots). *Bottom right*: Hydration shell densities determined by this procedure, for which the experimental and theoretical R_G match the best (opaque symbols and lines; "Scoring against R_G"). As a comparison the hydration shell densities, obtained from "standard" fits against the entire experimental SAXS curves (i.e. by using the hydration shell as a fit parameter), are equally plotted (semitransparent symbols and lines; "CRYSOL standard").

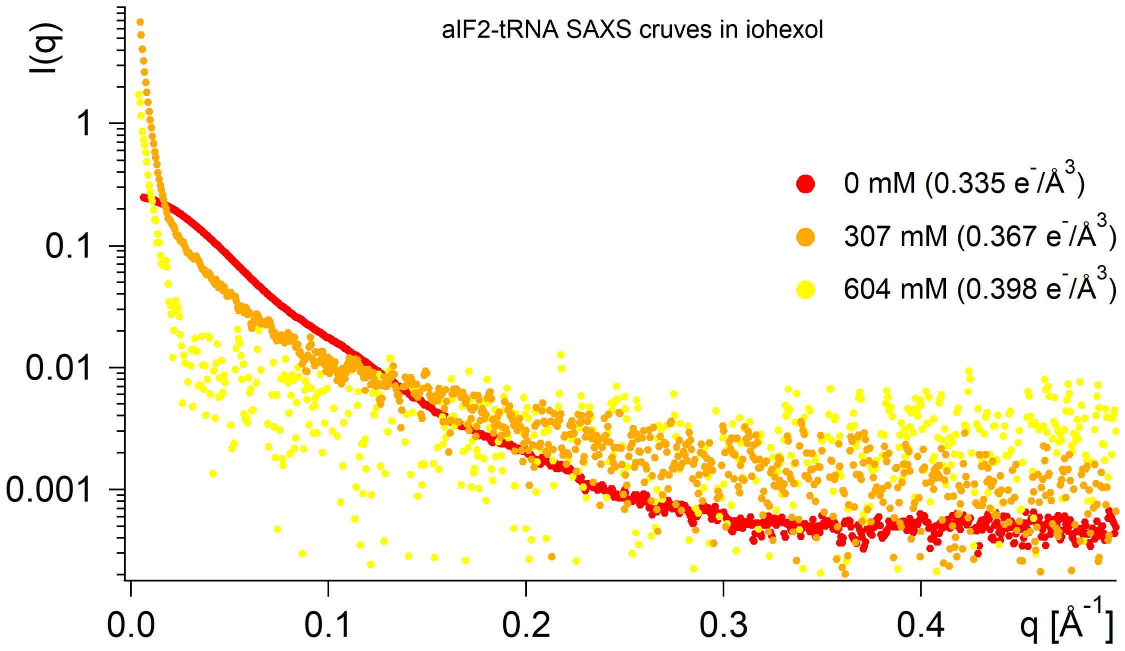

Figure S4 aIF2-tRNA SAXS curves at three different iohexol concentrations. The very strong increase of intensities scattered at low angles reveals aggregation of the complex at 307 and 604 mM iohexol, and no structural analysis was attempted.

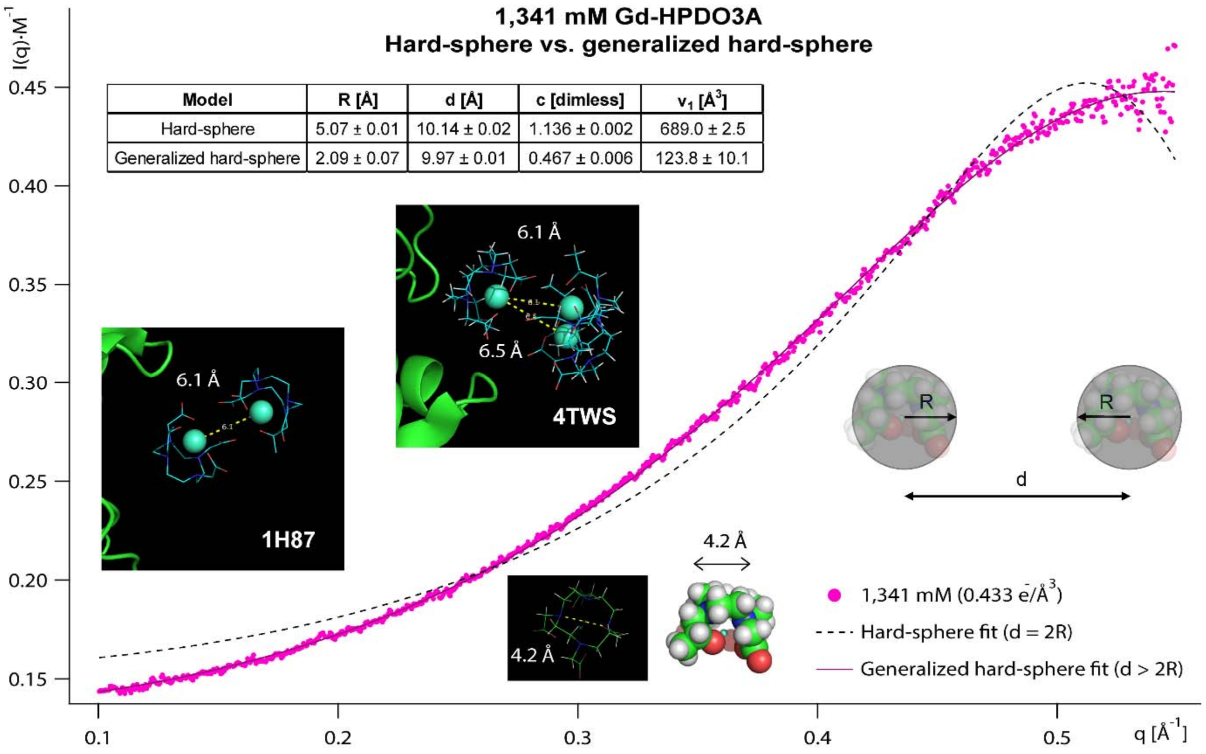

Figure S5 Inter-particle fits of 1341 mM Gd-HPO3A data. Fit curves and parameters of a classical hard-sphere and a generalized hard-sphere models (Eq. S4). *R* corresponds to the radius of a homogeneous sphere with constant contrast, and *d* to the distance between two sphere centers. A classical hard-sphere model ($d = 2R$) was not able to fit the data correctly (broken line), while relaxing the condition (i.e. allowing $d > 2R$) yielded a very satisfactory fit (continuous line). *Inset*: dimensions of Gd-HPDO3A molecule (bottom), and crystallographic models of pairs of Gd-HPDO3A molecules in the vicinity of lysozyme (PDB IDs 1H87 (Girard *et al.*, 2002) and 4TWS (Holton *et al.*, 2014)). 4.2 Å corresponds to the distance between two opposite nitrogen atoms in the macrocyle of a single HPDO3A molecule.

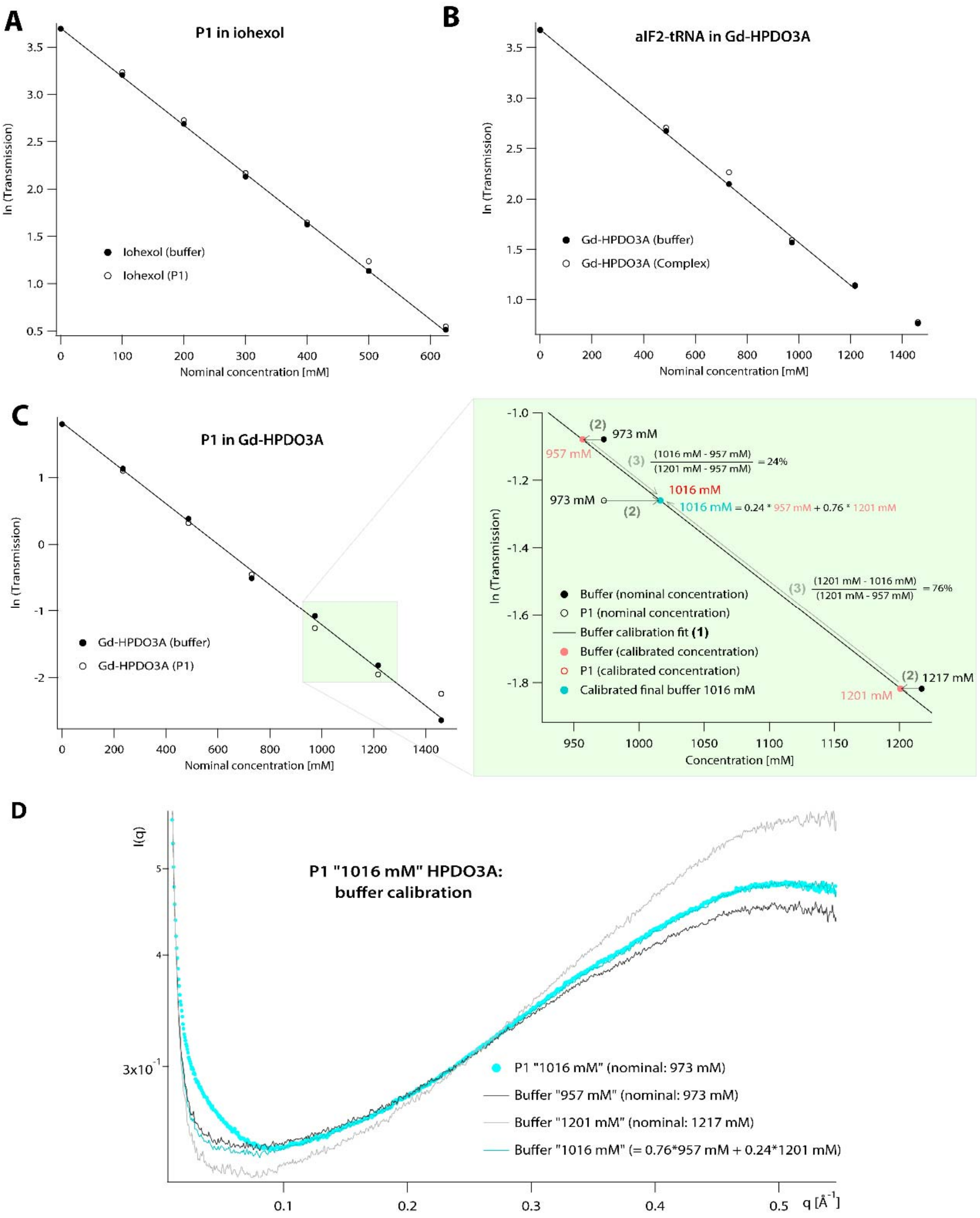

Figure S6 Concentration calibrations and buffer correction protocol. Due to strong inter-particle effects, the Gd-HPDO3A and iohexol buffer levels were sensitive to small variations of the exact concentrations, and needed to be matched to the respective sample curves. (A-C) Logarithm of buffer transmissions as a function of contrast agent concentrations, and linear fits used for calibration. (C, green inset) Zoom between 940 and 1220 mM Gd-HPDO3A to illustrate the individual steps of the buffer correction protocol: (1) Generation of a buffer calibration curve by a linear fit of the logarithm of the transmissions against nominal concentrations (see also (A-C)). (2) The real concentrations of buffers and samples are determined by comparing their transmissions with those of the calibrated curve. (3) Matching of the buffer level to the baseline of the corresponding sample by a linear interpolation of the two nearest buffer curves above and below the sample (see example in (D)). (D) Sample and buffer curves (initial and corrected) from example (C).

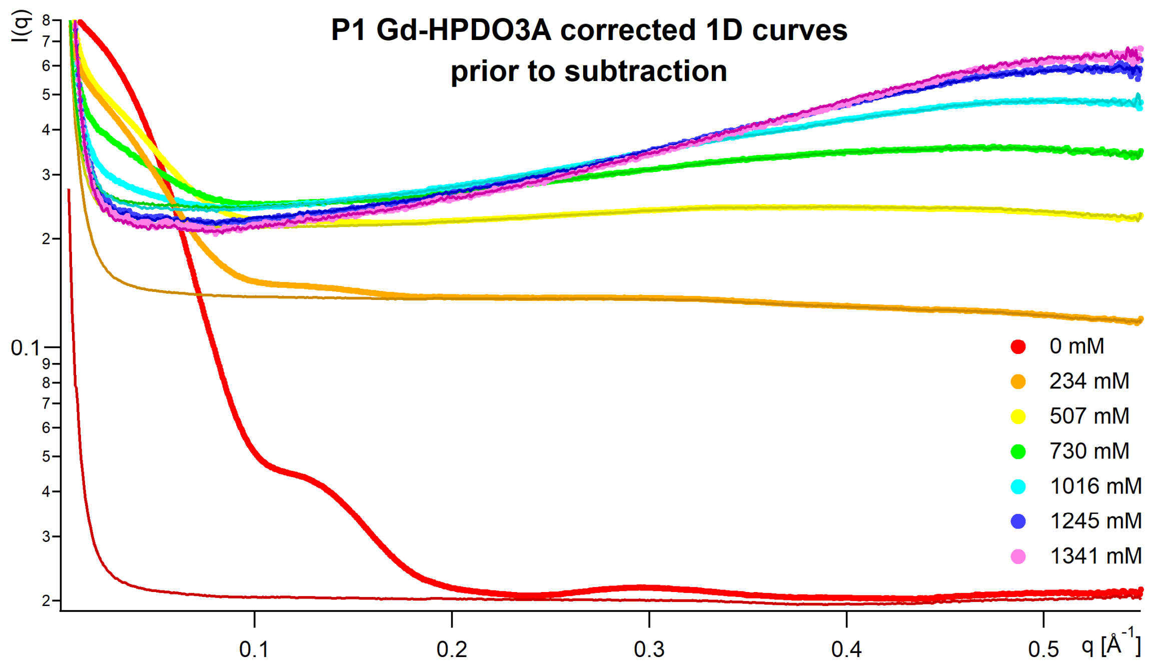

Figure S7 P1 SAXS curves in Gd-HPDO3A and corrected buffers. Concentrations of all samples and buffers were calibrated against transmissions, and buffers were matched to their respective samples by a linear interpolation of adjacent buffers (Fig. S6). SAXS curves of free Gd-HPDO3A (Fig. 6 and S5) were obtained from a subtraction of the 0 mM buffer (thin red line) from the buffers in the presence of Gd-HPDO3A (colored thin lines).

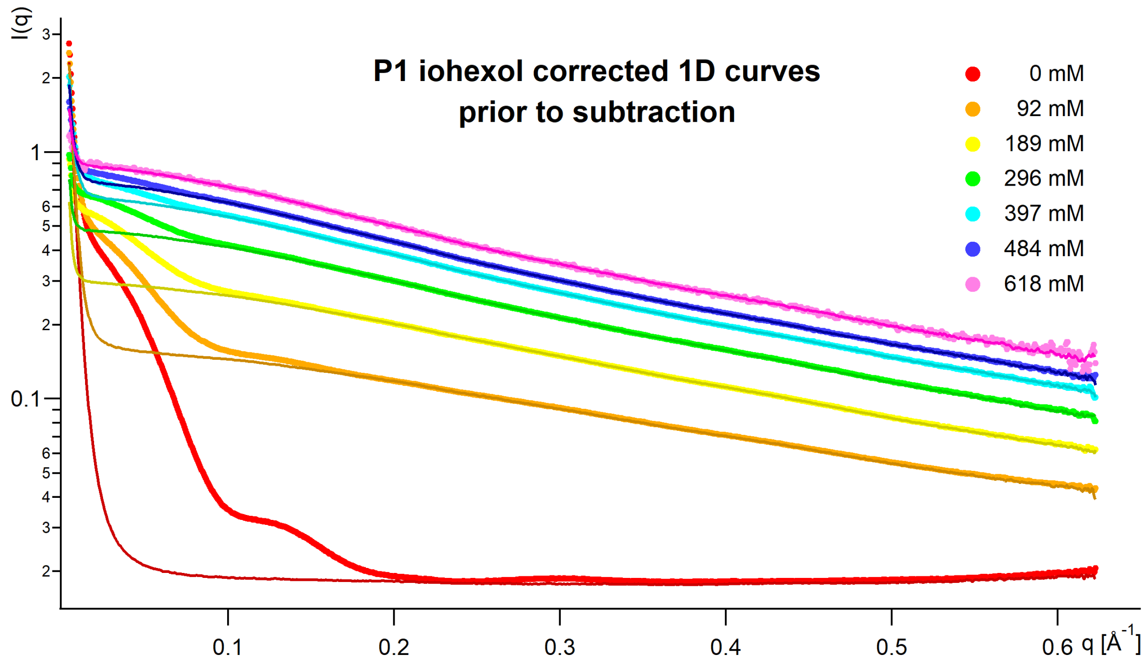

Figure S8 P1 SAXS curves in iohexol and corrected buffers. Concentrations of all samples and buffers were calibrated against transmissions, and buffers were matched to their respective samples by a linear interpolation of adjacent buffers (Fig. S6). SAXS curves of free iohexol (Fig. 5) were obtained by a subtraction of the 0 mM buffer (thin red line) from the buffers in the presence of iohexol (thin colored lines).

P1:

MKVLFLTANEFEDVELIYPYHRLKEEGHEVYIASFERGTITGKHGYSVKVDLTFDKVNPEEFD
ALVLPGGRAPERVRLNEKAVSIARKMFSEGKPVASICHGPQILISAGVLRGRKGTSFPGIKDD
MINAGVEWVDAEVVVDGNWVSSRVPADLYAWMREFVKLLK

aIF2-alpha D3 + gamma:

MRKVKMSGLITVRTNEPLGVEKIKEVISKALENIEQDYESLLNIKIYTIGAPRYRVDVVGTNPK
EASEALNQIISNLIKIGKEENVDISVVKK
AWPKVQPEVNIGVVGHVDHGKTTLVQAITGIWTSKHSEELKRGMTIKLGYAETNIGVCESCK
KPEAYVTEPSCKSCGSDDEPKFLRRISFIDAPGHEVLMATMLSGAALMDGAILVVAANEPFPQ
PQTREHFVALGIIGVKNLIIVQNKVDVVSKEEALSQYRQIKQFTKGTWAENVPIIPVSALHKINI
DSLIEGIEEYIKTPYRDLSQKPVMLVIRSFDVNKPGTQFNELKGGVIGGSIIQGLFKVDQEIKVL
PGLRVEKQGKVSYEPIFTKISSIRFGDEEFKEAKPGGLVAIGTYLDPSLTKADNLLGSIITLADA
EVPVLWNIRIKYNLLERVVGAKEMLKVDPIRAKETLMLSVGSSTTLGIVTSVKKDEIEVELRR
PVAVWSNNIRTVISRQIAGRWRMIGWGLVEI

tRNAfMet:

AGCGGGG(4SU)GGAGCAGCCUGG(H2U)AGCUCGUCGGG(OMC)UCAUAACCCGAAGAUC
GUCGG(5MU)(PSU)CAAAUCCGGCCCCCGCUACCA-methionine

Figure S9 Sequences of P1 and aIF2-tRNA.

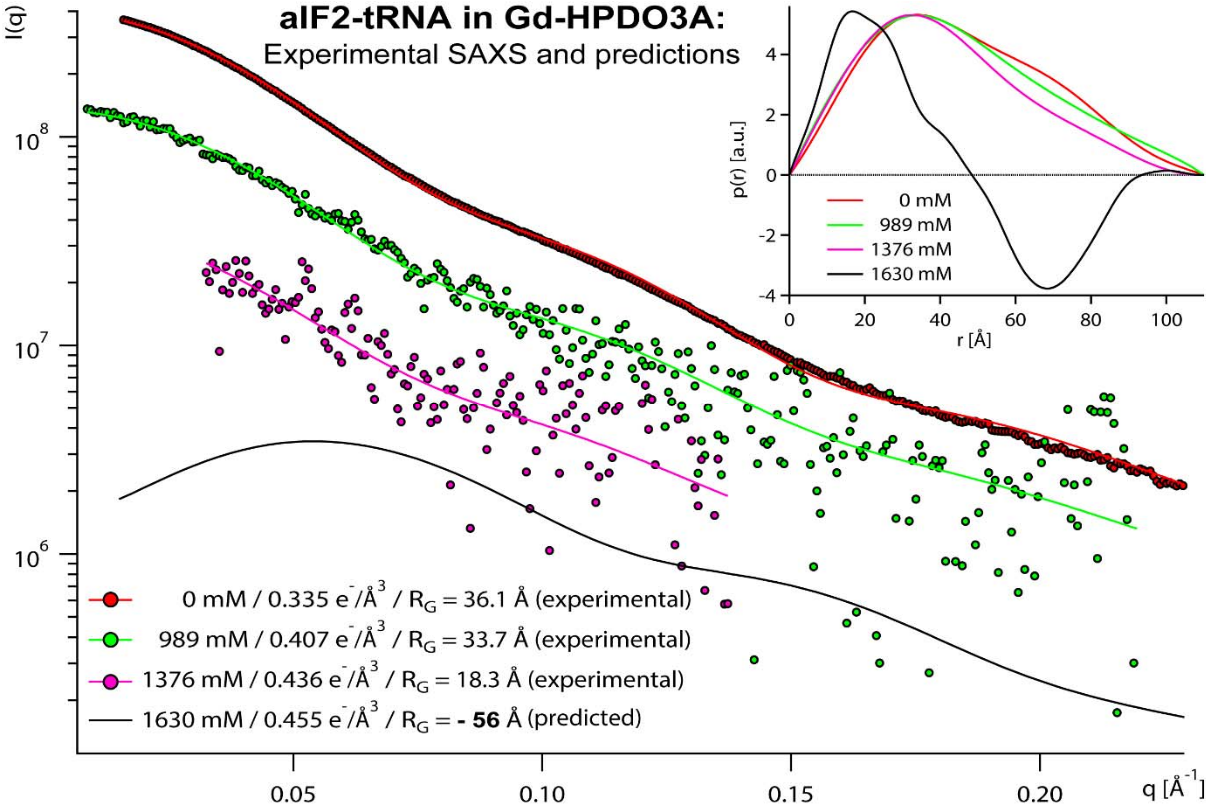

Figure S10 SAXS curves and *p*(*r*) function of the aIF2-tRNA complex: experiment and predictions. Illustration of experimental SAXS curves with CRYSOL fits and a CRYSOL-predicted SAXS curve of the protein-RNA complex at a higher solvent electron densities than in the present study. Such solvent densities could be reached by increasing the Gd-HPDO3A solubility by 15 % or by replacing the central Gd ion by Bi. p(r) curves were obtained by GNOM and then normalized to the highest values. Note that (apparent) negative R_G and negative values of p(r) are expected close to the CMP of a particle with zones of positive (RNA) and negative (protein) contrast, as routinely observed in small angle neutron scattering (Appolaire *et al.*, 2014).

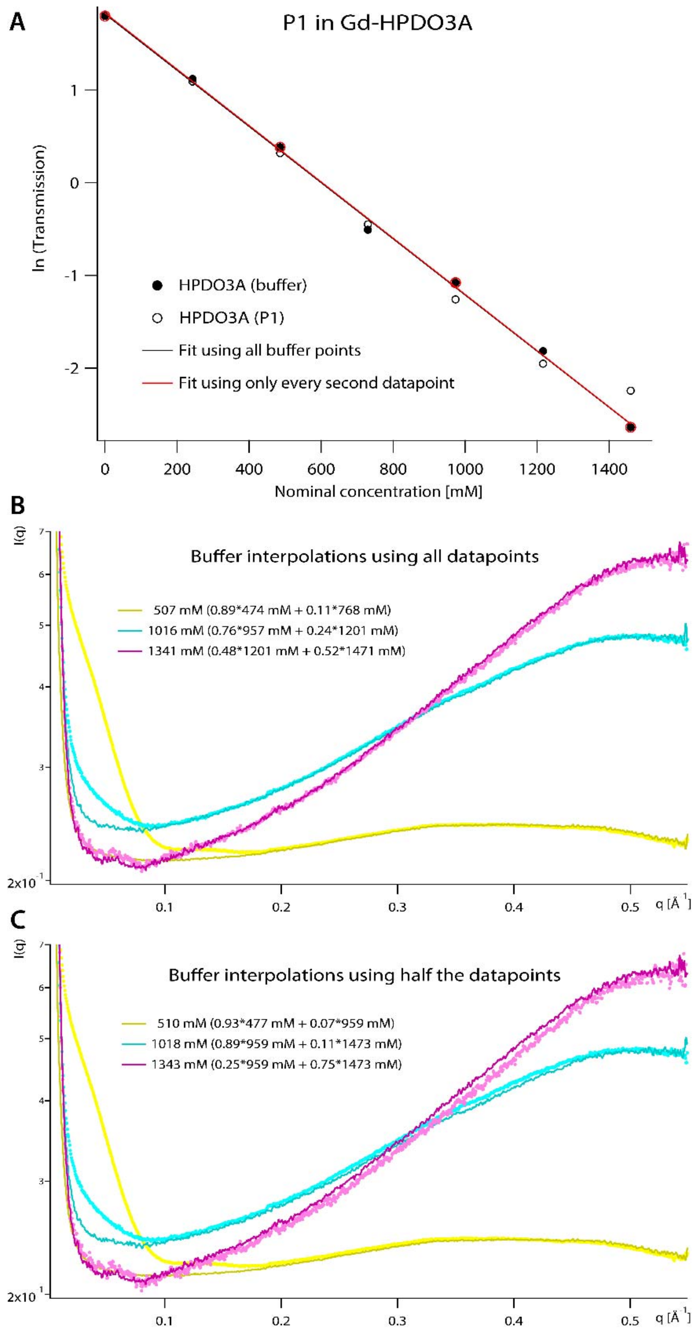

Figure S11 Stability and accuracy of buffer interpolation protocol in the case of P1 in Gd-HPDO$_3$A. This figure illustrates the precision and limits of the linear buffer interpolation protocol applied in the present work (see also Fig. S6) by comparing results from using the full range of data points measured with taking only every second data point into account. (A) Linear fits of the logarithmic transmissions used to calibrate the buffers. Black symbols: fit using all 7 data points. Red symbols: fit using only 4 data points. (B) The 1D sample and the respective aligned buffers at about 500, 1000 and 1500 mM when using an interpolation based on all data points (nearest neighbors about ± 250 mM). (C) The 1D sample and the respective aligned buffers at about 500, 1000 and 1500 mM when using an interpolation based on only half the data points (nearest neighbors about ± 500 mM).

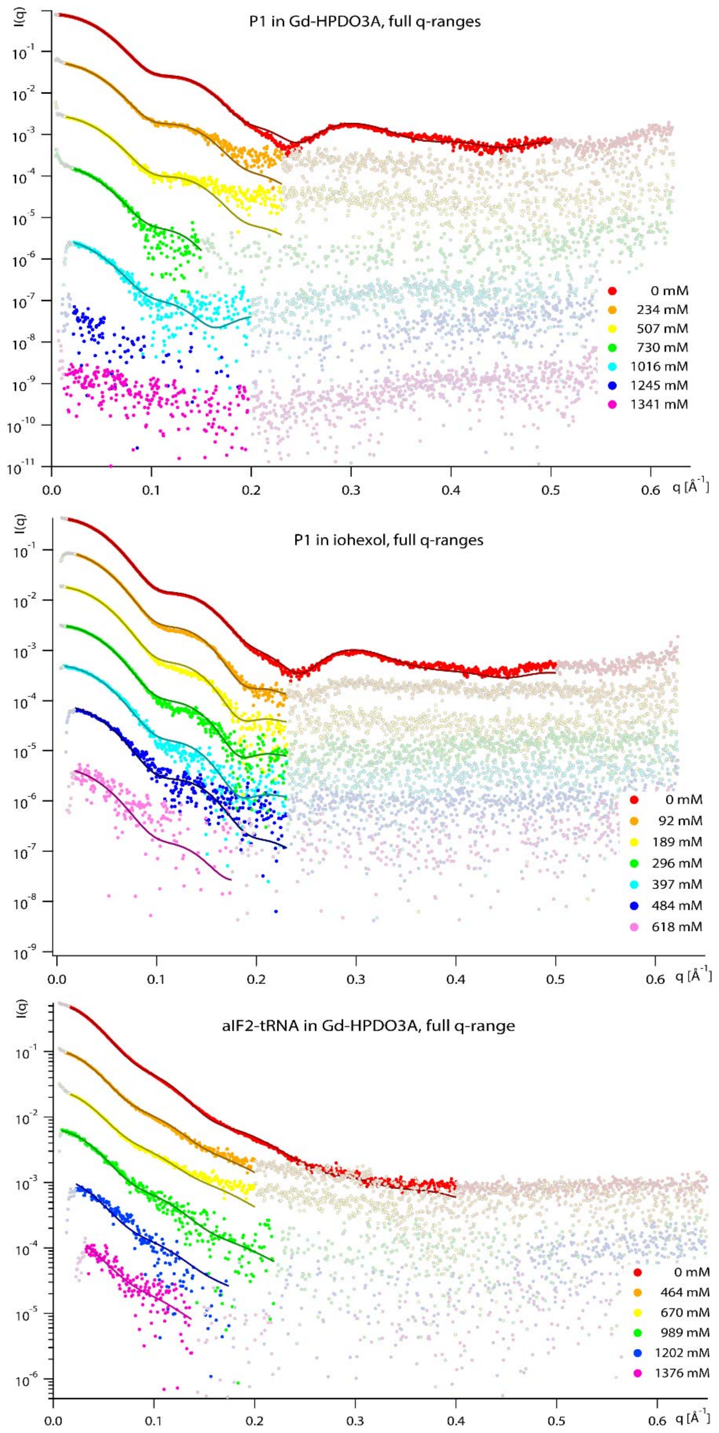

Figure S12 Full ranges of SAXS data recorded on P1 and aIF2-tRNA in Gd-HPDO$_3$A and P1 in iohexol. This figure presents the data and fits shown in Figure 1-3 B as opaque, supplemented by the data points (shown in semi-transparent mode) at very low angles and at high angles that were discarded and not used for the data analysis, due to artifacts (buffer mismatch, parasitic scattering at low angles etc). All data points with negative intensities (mainly at high angles due to buffer over-subtraction) are not included.

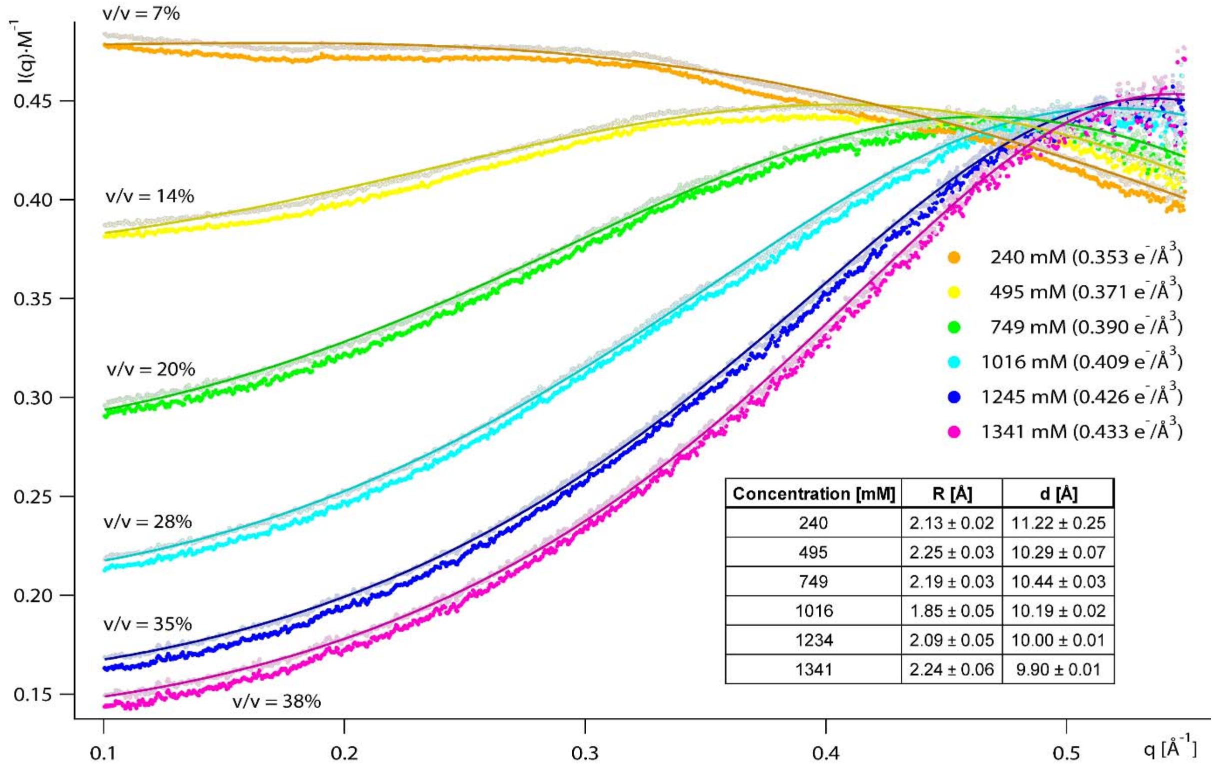

Concentration [mM]	R [Å]	d [Å]
240	2.13 ± 0.02	11.22 ± 0.25
495	2.25 ± 0.03	10.29 ± 0.07
749	2.19 ± 0.03	10.44 ± 0.03
1016	1.85 ± 0.05	10.19 ± 0.02
1234	2.09 ± 0.05	10.00 ± 0.01
1341	2.24 ± 0.06	9.90 ± 0.01

Figure S13 Impact of buffer subtraction taking volume fraction into account. This figure compares isolated Gd-HPDO$_3$A data, subtracted with the 0 mM buffer without taking the volume fraction v/v occupied by the contrast molecules into account (opaque points, identical to the data shown in Fig. 6). The transparent points show the subtracted curves with a weighted 0 mM buffer subtraction, diminished by the volume fraction that the contrast molecules occupy (calculated from Table S2). The inset table shows the fit parameters with this weighted SAXS curves. The fit values shown here are very similar to the ones in Figure 6, obtained for the non-weighted buffer subtraction, typically within 1% (with an exception of the data at 1341 mM).

References

Afonine, P. V., Grosse-Kunstleve, R. W., Echols, N., Headd, J. J., Moriarty, N. W., Mustyakimov, M., Terwilliger, T. C., Urzhumtsev, A., Zwart, P. H. & Adams, P. D. (2012). *Acta Crystallographica Section D* **68**, 352-367.

Aggerbeck, L., Yates, M., Tardieu, A. & Luzzati, V. (1978). *Journal of Applied Crystallography* **11**, 466-472.

Ajito, S., Hirai, M., Iwase, H., Shimizu, N., Igarashi, N. & Ohta, N. (2018). *Physica B: Condensed Matter* **551**, 249-255.

Appolaire, A., Girard, E., Colombo, M., Dura, M. A., Moulin, M., Haertlein, M., Franzetti, B. & Gabel, F. (2014). *Acta Crystallogr D Biol Crystallogr* **70**.

Ballauff, M. (2001). *Current Opinion in Colloids & Interface Science* **6**, 132-139.

Bolze, J., Ballauff, M., Kijlstra, J. & Rudhardt, D. (2003). *Macromol. Mater. Eng.* **288**, 495-502.

Bolze, J., Hoerner, K. D. & Ballauff, M. (1996). *Langmuir* **12**, 2906-2912.

Breyton, C., Gabel, F., Lethier, M., Flayhan, A., Durand, G., Jault, J.-M., Juillan-Binard, C., Imbert, L., Moulin, M., Ravaud, S., Härtlein, M. & Ebel, C. (2013). *The European Physical Journal E* **36**, 71.

Brosey, C. A. & Tainer, J. A. (2019). *Current Opinion in Structural Biology* **58**, 197-213.

Caravan, P., Ellison, J. J., McMurry, T. J. & Lauffer, R. B. (1999). *Chemical reviews* **99**, 2293-2352.

Chen, Y., Tokuda, J. M., Topping, T., Meisburger, S. P., Pabit, S. A., Gloss, L. M. & Pollack, L. (2017). *Proc Natl Acad Sci U S A* **114**, 334-339.

Creighton, T. E. (1993). *Proteins: Structures and Molecular Properties*. New York: W.H. Freeman and Company.

David, G. & Perez, J. (2009). *Journal of Applied Crystallography* **42**, 892-900.

Dingenouts, N. & Ballauff, M. (1993). *Acta Polymer.* **44**, 178-183.

Du, X., Choi, I.-G., Kim, R., Wang, W., Jancarik, J., Yokota, H. & Kim, S.-H. (2000). *Proceedings of the National Academy of Sciences* **97**, 14079.

Dubiez, E., Aleksandrov, A., Lazennec-Schurdevin, C., Mechulam, Y. & Schmitt, E. (2015). *Nucleic Acids Research* **43**, 2946-2957.

Emsley, P., Lohkamp, B., Scott, W. G. & Cowtan, K. (2010). *Acta Crystallographica Section D* **66**, 486-501.

Engilberge, S., Riobé, F., Di Pietro, S., Lassalle, L., Coquelle, N., Arnaud, C.-A., Pitrat, D., Mulatier, J.-C., Madern, D., Breyton, C., Maury, O. & Girard, E. (2017). *Chemical Science* **8**, 5909-5917.

Feigin, L. A. & Svergun, D. I. (1987). *Structure Analysis by Small-Angle X-Ray and Neutron Scattering*. New York and London: Plenum Press.

Fernandez, R. M., Riske, K. A., Amaral, L. Q., Itri, R. & Lamy, M. T. (2008). *Biochim Biophys Acta* **1778**, 907-916.

Fournet, G. (1951). *Acta Crystallographica* **4**, 293-301.

Franke, D., Petoukhov, M. V., Konarev, P. V., Panjkovich, A., Tuukkanen, A., Mertens, H. D. T., Kikhney, A. G., Hajizadeh, N. R., Franklin, J. M., Jeffries, C. M. & Svergun, D. I. (2017). *Journal of Applied Crystallography* **50**, 1212-1225.

Gabel, F. (2015). *Methods Enzymol* **558**, 391-415.

Gabel, F., Engilberge, S., Perez, J. & Girard, E. (2019). *IUCrJ* **6**, 521-525.

Garcia-Diez, R., Sikora, A., Gollwitzer, C., Minelli, C. & Krumrey, M. (2016). *Eur. Polymer J.* **81**, 641-649.

Girard, E., Chantalat, L., Vicat, J. & Kahn, R. (2002). *Acta Crystallographica Section D* **58**, 1-9.

Glatter, O. (2018). *Scattering Methods and their Application in Colloid and Interface Sciences*. Elsevier.

Grishaev, A., Anthis, N. J. & Clore, G. M. (2012). *J Am Chem Soc* **134**, 14686-14689.

Guinier, A. (1939). *Annales de Physique (Paris)* **12**, 161-236.

Guinier, A. & Fournet, G. (1955). *Small Angle Scattering of X-rays*. New York: John Wiley & Sons.

Haertlein, M., Moulin, M., Devos, J. M., Laux, V., Dunne, O. & Trevor Forsyth, V. (2016). *Methods in Enzymology*, edited by Z. Kelman, pp. 113-157: Academic Press.

Hajizadeh, N. R., Franke, D. & Svergun, D. I. (2018). *Journal of Synchrotron Radiation* **25**, 906-914.

Hartl, C., Frank, K., Amenitsch, H., Fischer, S., Liedl, T. & Nickel, B. (2018). *Nano Letters* **18**, 2609-2615.

Henke, B. L., Gullikson, E. M. & Davis, J. C. (1993). *Atomic Data and Nuclear Data Tables* **54**, 181-342.

Hickl, P., Ballauff, M. & Jada, A. (1996). *Macromolecules* **29**, 4006-4014.

Holton, J. M., Classen, S., Frankel, K. A. & Tainer, J. A. (2014). *The FEBS Journal* **281**, 4046-4060.

https://www.synchrotron-soleil.fr/en/beamlines/swing.

Jacrot, B. (1976). *Reports on Progress in Physics* **39**, 911-953.

Jeffries, C. M., Graewert, M. A., Blanchet, C. E., Langley, D. B., Whitten, A. E. & Svergun, D. I. (2016). *Nat Protoc* **11**, 2122-2153.

Jeffries, C. M., Ilavsky, J., Martel, A., Hinrichs, S., Meyer, A., Pedersen, J. S., Sokolova, A. V. & Svergun, D. I. (2021). *Nature Reviews Methods Primers* **1**, 70.

Kabsch, W. (2010). *Acta Crystallographica Section D* **66**, 125-132.

Kharakoz, D. P. (1997). *Biochemistry* **36**, 10276-10285.

Kikhney, A. G., Borges, C. R., Molodenskiy, D. S., Jeffries, C. M. & Svergun, D. I. (2020). *Protein Science* **29**, 66-75.

Kirste, R. G. & Stuhrmann, H. B. (1967). Elimination der intrapartikulären Untergrundstreuung bei der Röntgenkleinwinkelstreuung an kompakten Teilchen, Vol. 56, Zeitschrift für Physikalische Chemie, p. 338.

Kiselev, A. M., Lesieur, P., Kisselev, A. M., Lombardo, D., Killany, M. & Lesieur, S. (2001). *Journal of Alloys and Compounds* **328**, 71-76.

Kiselev, M. A., Wartewig, S., Janich, M., Lesieur, P., Kiselev, A. M., Ollivon, M. & Neubert, R. (2003). *Chem Phys Lipids* **123**, 31-44.

Knight, C. J. & Hub, J. S. (2015). *Nucleic Acids Research* **43**, W225-W230.

Lindner, P. & Zemb, T. (2002a). *Neutron, X-rays and Light: Scattering Methods Applied to Soft Condensed Matter*. Amsterdam, the Netherlands: Elsevier.

Lindner, P. & Zemb, T. (2002b). *Neutrons, X-rays and Light: Scattering Methods Applied to Soft Condensed Matter*. Elsevier.

Lipfert, J. & Doniach, S. (2007). *Annual Review of Biophysics and Biomolecular Structure* **36**, 307-327.

Madl, T., Güttler, T., Görlich, D. & Sattler, M. (2011). *Angewandte Chemie International Edition* **50**, 3993-3997.

Mahieu, E. & Gabel, F. (2018). *Acta Crystallographica Section D* **74**, 715-726.

Moelbert, S., Normand, B. & De Los Rios, P. (2004). *Biophysical Chemistry* **112**, 45-57.

Monestier, A., Aleksandrov, A., Coureux, P.-D., Panvert, M., Mechulam, Y. & Schmitt, E. (2017). *RNA* **23**, 673-682.

Naruse, K., Eguchi, K., Akiba, I., Sakurai, K., Masunaga, H., Ogawa, H. & Fossey, J. S. (2009). *J Phys Chem B* **113**, 10222-10229.

Putnam, C. D., Hammel, M., Hura, G. L. & Tainer, J. A. (2007). *Quarterly Reviews of Biophysics* **40**, 191-285.

Schmitt, E., Panvert, M., Lazennec-Schurdevin, C., Coureux, P.-D., Perez, J., Thompson, A. & Mechulam, Y. (2012). *Nature Structural & Molecular Biology* **19**, 450-454.

Sinibaldi, R., Ortore, M. G., Spinozzi, F., Carsughi, F., Frielinghaus, H., Cinelli, S., Onori, G. & Mariani, P. (2007). *The Journal of Chemical Physics* **126**, 235101.

Stuhrmann, H. (1970). *Zeitschrift fuer Physikalische Chemie* **72**, 185-198.

Svergun, D. I. (1992). *Journal of Applied Crystallography* **25**, 495-503.

Svergun, D. I., Barberato, C. & Koch, M. H. J. (1995). *Journal of Applied Crystallography* **28**, 768-773.

Svergun, D. I., Koch, M. H. J., Timmins, P. A. & May, R. P. (2013). *Small Angle X-ray and Neutron Scattering from Solutions of Biological Macromolecules*. Oxford: Oxford University Press.

Svergun, D. I., Richard, S., Koch, M. H. J., Sayers, Z., Kuprin, S. & Zaccai, G. (1998). *Proceedings of the National Acadamy of Sciences USA* **95**, 2267-2272.

Thureau, A., Roblin, P. & Perez, J. (2021). *Journal of Applied Crystallography* **54**, 1698-1710.

Timasheff, S. N. (2002). *Biochemistry* **41**, 13473-13482.

Trewhella, J., Duff, A. P., Durand, D., Gabel, F., Guss, J. M., Hendrickson, W. A., Hura, G. L., Jacques, D. A., Kirby, N. M., Kwan, A. H., Perez, J., Pollack, L., Ryan, T. M., Sali, A., Schneidman-Duhovny, D., Schwede, T., Svergun, D. I., Sugiyama, M., Tainer, J. A., Vachette, P., Westbrook, J. & Whitten, A. E. (2017). *Acta Crystallographica Section D* **73**, 710-728.

Tuukkanen, A. T., Spilotros, A. & Svergun, D. I. (2017). *IUCrJ* **4**, 518-528.

Voss, N. R. & Gerstein, M. (2005). *Journal of Molecular Biology* **346**, 477-492.

Winn, M. D., Ballard, C. C., Cowtan, K. D., Dodson, E. J., Emsley, P., Evans, P. R., Keegan, R. M., Krissinel, E. B., Leslie, A. G. W., McCoy, A., McNicholas, S. J., Murshudov, G. N., Pannu, N. S., Potterton, E. A., Powell, H. R., Read, R. J., Vagin, A. & Wilson, K. S. (2011). *Acta Crystallographica Section D* **67**, 235-242.

Wolf, A. V., Brown, M. G. & Prentiss, P. G. (1989). *CRC Handbook of Chemistry and Physics*, 70th ed. Boca Raton, Florida: CRC Press, Inc.

Zaccai, G. & Jacrot, B. (1983). *Annual Review of Biophysics and Biomolecular Structure* **12**, 139-157.
